# A Human Lin^−^ CD123^+^ CD127^low^ Population Endowed with ILC Features and Migratory Capabilities Contributes to Immunopathological Hallmarks of Psoriasis

**DOI:** 10.3389/fimmu.2017.00176

**Published:** 2017-03-02

**Authors:** Luz María Mora-Velandia, Octavio Castro-Escamilla, Andrés González Méndez, Cristina Aguilar-Flores, Martha Velázquez-Avila, María Isabel Tussié-Luna, Juan Téllez-Sosa, César Maldonado-García, Fermín Jurado-Santacruz, Eduardo Ferat-Osorio, Jesus Martínez-Barnetche, Rosana Pelayo, Laura C. Bonifaz

**Affiliations:** ^1^Unidad de Investigación Médica en Inmunoquímica Hospital de Especialidades Centro Médico Nacional Siglo XXI, Instituto Mexicano del Seguro Social, Mexico City, Mexico; ^2^Universidad Nacional Autónoma de México (UNAM), Mexico City, Mexico; ^3^Unidad de Investigación en Enfermedades Oncológicas, Hospital de Oncología, Centro Médico Nacional ‘Siglo XXI’, Mexico City, Mexico; ^4^División de Investigación, Facultad de Medicina, Universidad Nacional Autónoma de México (UNAM), Mexico City, Mexico; ^5^Unidad de Investigación en Virología y Cáncer, Hospital Infantil de México “Federico Gómez”, Mexico City, Mexico; ^6^Centro de Investigación Sobre Enfermedades Infecciosas, Instituto Nacional de Salud Pública, Cuernavaca, Morelos, Mexico; ^7^Centro Dermatológico “Dr. Ladislao de la Pascua”, Secretaria de Salud de la Ciudad de México, Mexico City, Mexico

**Keywords:** innate lymphoid cells, psoriasis, IL-3Rα, IL-17, SDF-1, CXCR4 axis, skin inflammation

## Abstract

Innate lymphoid cells (ILC) are members of a heterogeneous family with a lymphoid origin that mimics the T helper (Th) cytokine profile. ILC are involved in early effector cytokine-mediated responses during infections in peripheral tissues. ILC also play an important role in chronic skin inflammatory diseases, including psoriasis. Although classical ILC express CD127, it has been recently reported that the presence of non-classical CD127^−^ ILC populations and an early ILC precursor (EILP) CD127^low^. ILC development has predominately been investigated in mouse models. However, in humans, different transcription factors have been described for ILC identification. NFIL3 (nuclear factor, IL-3 regulated) is crucial for ILC development in response to IL-7. CD123 (IL-3Rα) is usually used to exclude basophils during ILC identification, however, it is unknown if in response to IL-3, NFIL3 could be relevant to induce ILC features in Lin^−^ CD123^+^ populations in addition, is also unknown whether peripheral blood (PB) population with ILC features may have skin-homing potential to participate in skin inflammatory chronic diseases. Here, we report a Lin^−^ CD123^+^ CD127^low^ CD7^+^ CLA^+^ population that share some phenotypic properties with basophils, but expresses several transcription factors for ILC commitment such as inhibitor of DNA binding 2 (Id2), NFIL3, promyelocytic leukemia zinc finger (PLZF), thymocyte selection-associated high-mobility group box protein (TOX), and T cell factor-1 (TCF-1). In addition, this population expresses different ILC markers: CD132, CD90, CD161, α4 integrin, c-Kit, CRTH2, AhR, and IL-23R. IL-3 prevents apoptosis and increases their NFIL3, TOX, and PLZF expression. In PB, the CD123^+^ CD127^low^ population is predominantly a conspicuous population that expresses T-bet and RORγt. The Lin^−^ CD123^+^ CD127^low^ population in PB has a limited Th type cytokine expression and highly expresses IL-8. The Lin^−^ CD123^+^ CD127^low^ population expresses skin-homing receptors (cutaneous lymphocyte antigen and CXCR4) and transmigrates through endothelial cells in response to SDF-1. An equivalent Lin^−^ CD123^low^ population was identified in control skin, which shows a broader phenotypic diversity and cytokine production, including IL-22 and IL-17. Remarkably, the CD123^low^ population in the lesion and non-lesion skin of psoriasis patients expresses IL-17 and IL-22. Our findings suggest the identification of an alternative Lin^−^ CD123^+^ CD127^low^ population with ILC features endowed with migratory capabilities that might contribute to immunopathological hallmarks of psoriasis.

## Introduction

Innate lymphoid cells (ILC) have been defined as a heterogeneous family derived from a CD7^+^ common lymphoid precursor (CLP) (1–3). During the previous decade, several ILC populations that participate in the defense against pathogens and inflammatory diseases have been described mainly in mice (4, 5). The identification of ILC populations in humans, as well as their role in disease pathogenesis, comprises a topic of extensive investigation.

Several groups have used distinct criteria and markers for ILC identification. ILC have a classical lymphoid morphology; they lack T cell receptor or BCR expression and are considered lineage negative (Lin^−^) cells. In humans, the most common lineage markers include CD3, CD19 (for T and B cells), CD14 (monocytes), and CD11c, as well as blood dendritic cell antigen (BDCA)-1, -2 (dendritic cells). CD123 expression has been used to exclude plasmacytoid dendritic cells (pDCs) and basophils, while FcεR for basophils and mast cells (6, 7).

Classical ILC in human peripheral blood (PB) express CD127. However, a non-classical category of ILC CD127^−^ (8) as well as an early ILC precursor (EILP) CD127^low^ (9) has been recently reported. In addition to CD127, other markers have been used in human PB for ILC identification, ILC express CD132 (γ common chain), which is crucial for development, as well as CD90 and CD161 as ILC markers (2). The expression of α4β7 integrin has been reported in ILC precursors in mice (10–12). In addition to surface markers, several transcription factors have been used for ILC identification. The transcription factor inhibitor of DNA binding 2 (Id2) is essential for identification and linage commitment (13, 14). In addition to Id2, transcriptional factors, such as thymocyte selection-associated high-mobility group box protein (TOX) and promyelocytic leukemia zinc finger (PLZF), have been described as essential for ILC development (15–17); in humans, these additional transcriptional factors have primarily been used as ILC lineage-related markers. Recently, T cell factor-1 (TCF-1) has been also described in mice as crucial for ILC development (18). Remarkably, it has been demonstrated that the nuclear factor, IL-3 regulated (NFIL3) is a crucial transcription factor in ILC development in response to IL-7 (19). Although CD123 (IL-3Rα) is usually used to exclude basophils and mast cells during ILC identification, it is unknown if in response to IL-3, NFIL3 might be relevant to induce ILC features in Lin^−^ CD123^+^ populations.

Based on the criteria used to identify ILC in mice, three main groups of ILC have been described and identified in humans (2, 20) as CD161^+^. In addition, ILC1 are c-Kit^−^ and CRTH2^−^ and express T-bet and IFN-γ, while ILC2 are c-Kit^+^ and CRTH2^+^ and express GATA-3, IL-4, IL-5, and IL-13, and ILC3 are c-Kit^+^, CRTH2^−^ are AhR^+^ (aryl hydrocarbon receptor), IL23R^+^ CCR6^+^ and express RORγt, IL-17, and IL-22 (6, 21, 22). ILC3 may also express natural killer (NK) receptors such as NKp44 (22, 23). It has been proposed that ILC mimic the effector function of T helper (Th) lymphocytes (Th1, Th2, and Th17). However, ILC activation is mainly mediated by cytokines expressed by other innate cells, such as dendritic cells, macrophages, or stromal cells (24, 25). In general, the most frequent *in vitro* conditions used to resemble the *in vivo* activation include the use of IL-12 and IL-15 or IL-18 for ILC1, IL-25, IL-33, and TSLP for ILC2 and IL-1β, IL-2 and IL-23 for ILC3 and, in some cases, the presence of IL-7 (26).

As a result of the increasing numbers of ILC studies, it has been possible to establish the presence and diversity of classical and, to a less extent, non-classical ILC populations in different peripheral tissues (27); mouse models have demonstrated that ILC together with other innate cells are the first line of defense against pathogens (28–31). Recently, a regulatory role for ILC populations have been reported (32). Therefore, in humans, there is increasing evidence that ILC play a role in several pathologies, such as allergies and chronic inflammatory skin disorders (33), including psoriasis (34, 35). Interestingly, the proportions of the different subsets (ILC1, ILC2, and ILC3) among tissues appear to be different, and it also appears that the local microenvironment may influence the “specialized” functions of ILC (36, 37). It has been proposed that ILC in PB may represent a reservoir of ILC in which their functional features may be distinct from peripheral tissues (7, 24, 38). Nevertheless, the mechanisms that underlie the migration of ILC into different tissues under steady state or inflammatory conditions are in the early stages of investigation. In particular, for skin migration, it has been reported that in PB, ILC2 and ILC3 express cutaneous lymphocyte antigen (CLA) (39, 40), which is the main assumed mechanism of ILC skin tropism under steady-state conditions; however, additional migration mechanisms under inflammatory conditions have not been established to date.

In the skin, one of the main human pathologies in which the participation of ILC has been investigated is psoriasis. It has been described that blood and skin samples from patients have increased ILC3 NCR^+^ frequencies (40, 41), and although the IL-22-producing ILC3 had been well identified, the production of IL-17 has been reported in lymphoid CD3^−^ cells. These findings suggest that in the skin, other cell populations (Lin^−^ CD45^+^ CD3^−^) exist that produce IL-17. Nevertheless, it has not been well established whether these cells are related to the ILC lineage.

Here, we identified a Lin^−^ CD123^+^ CD127^low^ population in the PB of healthy donors (HD) that express several ILC features and in which IL-3 appears to be essential for their maintenance and identity. Interestingly, this Lin^−^ CD123^+^ CD127^low^ population highly expresses CLA and exhibits migratory potential in response to SDF-1. Remarkably, a similar Lin^−^ CD123^low^ population was identified in control skin (CS) and importantly in psoriasis skin (PS) biopsies with the capability to express IL-22 and IL-17. These findings suggest that this population with ILC features may contribute to the immunopathological features of psoriasis.

## Materials and Methods

### Blood Sample Collection

Buffy coats of HD were obtained from the Blood Bank from the “Hospital Infantil de México: Federico Gómez.” Peripheral blood mononuclear cells (PBMCs) were isolated with Lymphoprep (Axis-Shield, Oslo, Norway) from buffy coats.

### Skin Biopsies from CS and Psoriasis Patients

Control skin was obtained from remnant skin following plastic or abdominal surgeries that was free from dermatologic pathologies from the “Hospital de Especialidades Dr. Bernardo Sepulveda CMN Siglo XXI.” Patients were recruited from the dermatology clinic of the *Centro Dermatológico Dr*. *Ladislao de la Pascua*. Fifteen patients who fulfilled the diagnostic criteria for psoriasis in plaque were included in this study prior to treatment initiation. Patient biopsies were obtained with a 5–6 mm punch.

### Skin Cell Collection

Skin samples were placed overnight in RPMI medium and dispase II (Grade II protease, Roche, Switzerland). The dermis was then mechanically separated from the epidermis. Dermal cells were obtained by allowing migration from dermal segments placed in culture in RPMI medium for 7 days. The collected cells were used for activation experiments, and the cell supernatants were used as chemoattractant stimuli in migration assays.

### Flow Cytometry Analysis and Sorting

In order to block Fc receptors, PBMCs were incubated with an in-house-made buffer containing 2% horse serum. Cells were stained with a cocktail of antibodies (complete list included in Supplementary Material). Fixation was performed using 4% paraformaldehyde (PFA). All antibodies were isotype-matched with their respective fluorophore. Intracellular assessment of cytokines and transcriptional factors were performed using Cytofix/Cytoperm (BD Biosciences, San Jose, CA, USA) or the Factor Fixation and Permeabilization Buffer Set (Biolegend, San Diego, CA, USA), respectively. Cells were incubated with antibodies for 30 min at room temperature. Before cell fixation, Hoechst 33342 staining assessed cell viability during assays. The samples were acquired using a FACS Canto (BD Biosciences, San Jose, CA, USA) and were analyzed with Flowjo software (Tree Star). For cell isolation, PBMCs were stained with PE-conjugated lineage marker antibodies. Lineage^+^ cells (CD3^+^, CD14^+^, CD19^+^, CD94^+^, and HLA-DR^+^) were depleted using anti-PE Microbeads and LD columns (Miltenyi-Biotec, BG, Germany). The cells were sorted from the lymphoid region and according to CD123 and CD127 expression using a FACS Aria II (BD Biosciences, San Jose, CA, USA).

#### Heat Map Construction

The median fluorescence intensities (MFs) were determined for each cell surface marker and each subpopulation; the minimum value reported (gray) corresponds to the isotype control (MF), and the maximum value reported (red) corresponds to the cell population with the highest MF value.

#### Imaging Flow Cytometry

The morphology of total pre-enriched Lin^−^ CD123^+^ cells was evaluated in PBMCs previous to depletion of Lineage^+^ cells (as described above) and stained with anti-CD123 and the nuclear dye DAPI (Thermo Fisher Scientific, MA, USA). Cells were acquired using the Amnis ImageStream Mark II and analyzed by the IDEAS^®^ software (Merck-Millipore, MA, USA).

### Quantitative RT-PCR

Total RNA was extracted from a pool of three different cell donors using RNeasy Mini Kit (Qiagen, Hilden, Germany), and cDNA was synthesized using Maxima First Strand cDNA Synthesis kit (Thermo Fisher Scientific, MA, USA), according to the manufacturer’s instructions. Quantitative gene expression for human IL7Rα or for the housekeeping human GAPDH gene was performed using Maxima Syber Green qPCR Master Mix (Thermo Fisher Scientific, MA, USA) and a Rotorgene Real Time PCR System (Qiagen, Hilden, Germany). Used primers: IL7Rα-Forward: 5′ AGG ATG AAA ACA AAT GGA CGC A 3′. IL7Rα-Reverse: 5′ CCT TTA AAA TAG TGA TCA GGG ATG G 3′. Size of cDNA product: 238 bp.

### Incomplete D_H_–J_H_ Rearrangements Analysis

Genomic DNA was extracted from sorted cell populations using the QIAamp DNA Mini Kit (Qiagen, Hilden, Germany). The identification of incomplete D_H_–J_H_ rearrangements was performed using the BIOMED-2 primer sets as described (42). Briefly, two independent PCR reactions per DNA sample were set: a multiplexed PCR reaction combining a single J_H_ consensus primer and six D_H_ primers (Tube D) corresponding to six of the seven D_H_ segment families. The second reaction contained the same J_H_ primer and a single D_H_7 primer (Tube E). Each reaction product was subjected to capillary electrophoresis using the Agilent DNA 1000 chip in the Agilent 2100 Bioanalyzer system.

### Cell Activation

Peripheral blood mononuclear cells or skin cells were stimulated using the cell stimulation cocktail and the protein transport inhibition cocktail (for the last 6 h of culture) (eBioscience-Affymetrix, Santa Clara, CA, USA) to assess the production of cytokines via intracellular staining. When indicated, IL-3 was added to the culture for 18 h to evaluate the phenotype and expression of transcriptional factors. Furthermore, ILC1, ILC2, and ILC3 cocktails (Supplementary Material) were added to the culture for 18 h to determine the expression of intracellular cytokines; IL-3 and IL-7 were included in all cocktails.

#### IgE Crosslinking Assay

Peripheral blood mononuclear cells were incubated with 1 µg/mL of human IgE (Merck-Millipore, MA, USA) during 2 h, washed, and incubated with 2 µg/mL of anti-human IgE (BD Biosciences, San Jose, CA, USA) during 30 min; the activation of cells was assessed by the expression of phenotypic markers at the end of incubation.

### Immunofluorescence

Goat anti-Id2 (R&D Systems, Minneapolis, MN, USA), Biotin anti-goat IgG, Isotype control, AlexaFluor 488 Streptavidin (Jackson-lmmunoresearch, West Grove, CA, USA) and Vectashield-DAPI (Vector-laboratories, Burlingame, CA, USA) were used to stain sorted populations. Images were acquired using a Leica TCS SP8x Confocal Microscope (Wetzlar, Germany) and were analyzed with Leica software.

### Cell Viability Assay

Isolated Lin^−^ CD123^+^ CD127^low^ cells were cultured in the presence or absence of IL-3 (PeproTech, NJ, USA) for 3 days. Cells were collected and stained using the FITC Annexin V Apoptosis Detection Kit (BD Biosciences, San Jose, CA, USA) according to the manufacturer’s instructions. Samples were acquired using a FACS Canto (BD Biosciences, San Jose, CA, USA) and were analyzed with Flowjo software (Tree Star).

### Cytokine Quantification

Isolated Lin^−^ CD123^+^ CD127^low^ from the lymphoid region or Lin^−^ CD127^+^ cells were cultured in the presence or absence of IL-3 (PeproTech, NJ, USA) for 18 h ± stimulation cocktail (eBioscience-Affymetrix, Santa Clara, CA, USA) during the last 6 h of culture. The supernatants were collected and stored (−80°C) until analysis. The quantification was performed using a personalized (Supplementary Material) Magnetic Luminex Screening Assay (R&D Systems, Minneapolis, MN, USA). Sample acquisition and analysis were performed in a MagPix instrument (Luminex, TX, USA).

### Migration Assay in Transwell System

Using a transwell system (Corning, NY, USA), migration in response to stimuli, SDF-1 (R&D systems, Minneapolis, MN, USA) or supernatants from CS or PS cell cultures was assessed after 3 h. Migration was also assessed in the presence of blocking anti-CXCR4 (Biolegend, San Diego, CA, USA). The transmigration assays using endothelial cells, the upper chamber of the transwell was covered with attachment factor (Gibco, Thermo Scientific, MA, USA) during 1 h/37°. Human umbilical vein endothelial cells were cultured for 1–2 days (with supplemented EGM-2 medium, Lonza, Switzerland) in the previous covered well to promote monolayer formation. The monolayer was subsequently washed with PBS 1× and used for the migration assay. 2 × 10^6^ PBMCs per milliliter were placed in the upper chamber in the presence or absence of SDF-1 as stimuli to assess migration after 4 h. Cells from the upper and lower chambers were counted using a microscope and were analyzed by flow cytometry.

### Statistical Analysis

Statistical analysis was performed using the GraphPad Prism 5.0 (La Jolla, CA, USA) software. The non-parametric Mann–Whitney *U* test was used to calculate the statistical significance between the groups. All *p* values less than 0.05 were considered statistically significant.

## Results

### A Human Lin^−^ CD123^+^ CD127^low^ Cell Population with Lymphoid and Basophil Features in PB

**Figure 1:**
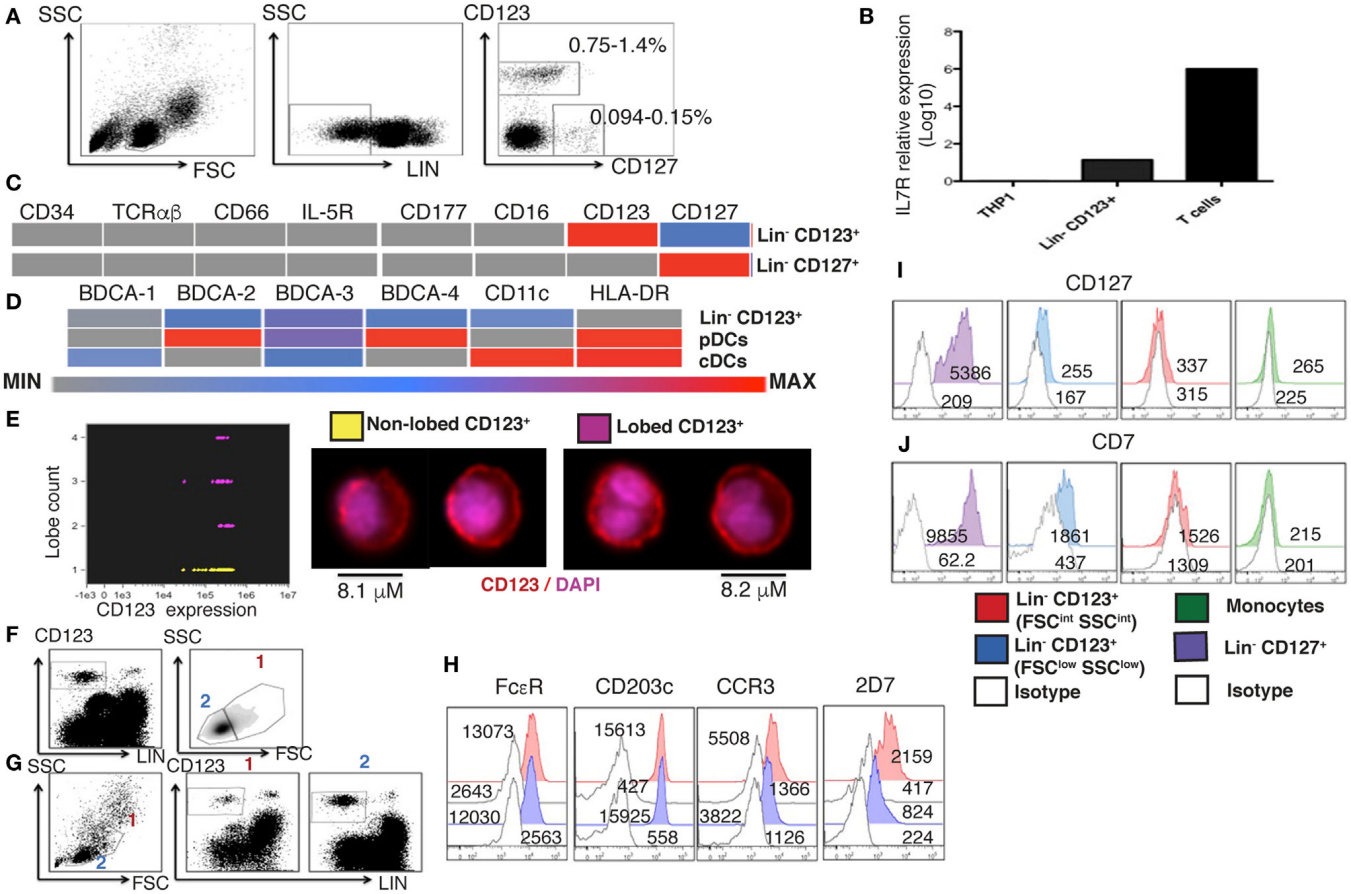
**A human Lin^−^ CD123^+^ CD127^low^ population with lymphoid and basophil features in peripheral blood**. **(A)** From left to right: gate analysis on lymphoid cells, exclusion of lineage positive cells and identification of Lin^−^ CD123^+^ and Lin^−^ CD127^+^ populations. Cell frequencies (%) data represent the media values from 30 human blood samples. **(B)** Lin^−^ CD123^+^ (isolated from the lymphoid region and gated as shown in Figure S1A in Supplementary Material) and T lymphocytes purified from a pool of three human blood samples by cell sorting were assessed for the expression of IL7Rα by quantitative RT-PCR. The monocyte cell line THP1 was used as negative control. Relative expression was normalized to GAPDH gene expression values. **(C,D)** Heat maps, representative of three independent experiments, are shown. Median fluorescence intensity (MF) values for the exclusion of additional lineage and dendritic cell markers, respectively. Color-coded scale is shown under the heat map from minimum (isotype) to maximum values. pDCs, plasmacytoid dendritic cells; cDCs, conventional dendritic cells. **(E)** Imaging flow cytometry analysis of total pre-enriched Lin^−^ CD123^+^ cells. The graph at the left shows CD123 expression vs. lobe count that categorizes enriched Lin^−^ CD123^+^ cells in to two populations: yellow: non-lobed cells and pink: lobed cells. At the right, the morphology of each population is shown (50 cells of each population were analyzed). **(F,G)** Compared cell analysis with two different gating strategies. **(F)** Lin^−^ CD123^+^ and further FSC vs. SSC, or **(G,H)** FSC vs. SSC followed by Lin^−^ vs. CD123^+^ gating. Both gating strategies showed the identification of two cell populations as FSC^int^ SSC^int^ (1) and FSC^low^ SSC^low^ (2) with a frequency of 0.3–1.1% and 0.7–1.3%, respectively, within total peripheral blood mononuclear cells (cell frequencies (%) represent media values of 10 human blood samples). **(H)** Basophil markers expression in both populations [gated as in panel **(G)**]. **(I,J)** CD127 and CD7 compared expression in different cell populations. Red: Lin^−^ CD123^+^ (FSC^int^ SSC^int^), blue: Lin^−^ CD123^+^ (FSC^low^ SSC^low^) [gated as in panel **(G)**], purple: Lin^−^ CD127^+^, green: monocytes, white: isotype for each cell population. MF values representative of three independent experiments are presented.

To determine the presence of Lin^−^ CD123^+^ (IL-3Rα) population in the PB of HD, cells with a lymphoid morphology and linage negative (Lin^−^) for T cells (CD3^−^), B cells (CD19^−^), monocytes (CD14^−^), NK cells (CD94^−^), and dendritic cells (HLA-DR^−^) were evaluated. Two distinct Lin^−^ cell populations were identified: the classical CD127^+^ population and a CD123^+^ with low expression of CD127 by both, flow cytometry and PCR (Figures 1A,B). The cell frequency of the CD123^+^ CD127^low^ population is approximately 0.7–1.4%, while the CD127^+^ population is approximately 0.09–0.15% of the total PBMC cells. The CD123^+^ CD127^low^ population is about 8–10 times more frequent than the CD127^+^ population (Figure 1A). As indicated in Figure 1C, both the Lin^−^ CD123^+^ CD127^low^ and CD127^+^ populations are negative for CD34, suggesting that they are not primitive precursor cells, as well as for T cells (TCRαβ), granulocytes (IL-5R, CD177, and CD66) and NK, monocytes, and neutrophil (CD16). The Lin^−^ CD123^+^ CD127^low^ was also negative for conventional dendritic cells (BDCA-1, BDCA-3, and CD11c) and pDCs (BDCA-2, BDCA-4, and HLA-DR) (Figure 1D; Figure S1 in Supplementary Material). As indicated in Figure S1 in Supplementary Material, the Lin^−^ CD123^+^ CD127^low^ population was absent when anti-FcεR was included in the linage cocktail. Therefore, in addition to CD123, we evaluated its morphology and expression of basophil markers, including FcεR, CCR3, CD203c, and an antigen expressed in the secretory granules of human basophils that is recognized by the monoclonal antibody 2D7 (43). Remarkably, imaging flow cytometry assay identified two populations of total pre-enriched Lin^−^ CD123^+^. As observed in Figure 1E, there is a mixture of lymphocyte cells with not segmented nuclei (no lobes), and cells with a classical basophil morphology (lobed nuclei). In addition, two cell populations by two different gating strategies were identified when Lin^−^ CD123^+^ and then FSC vs. SSC were analyzed (Figure 1F) or upon FSC vs. SSC and then Lin^−^ vs. CD123^+^ analysis (Figure 1G). These two cell populations were further defined as FSC^int^ SSC^int^ (1) and FSC^low^ SSC^low^ (2) with a frequency of 0.3–1.1 and 0.7–1.3%, within total PBMCs, respectively. Both populations showed similar expression of FcεR and CCR3. However, the expression of CD203c and the antigen identified by the 2D7 mAb are substantially lower in the CD123^+^ CD127^low^ population from the lymphoid region, consistent with its morphology (Figure 1H). To evaluate the possible lymphoid origin of the Lin^−^ CD123^+^ CD127^low^, we investigated the presence of incomplete D_H_–J_H_ rearrangements as a molecular fingerprint of early lymphoid precursors. As shown in Figures S2 and S3 in Supplementary Material, we could not find significant D_H_-J_H_ rearrangements in the CD127^+^ classical ILC or in the Lin^−^ CD123^+^ CD127^low^ population when compared with B-lymphocytes and acute B cell leukemia cell line NAL-M6. However, the expression of CD7 distinguished the lymphoid-related CD123^+^ cells from the CD123^+^ FSC^int^ SSC^int^ population and monocytes (Figures 1I,J). These findings indicate the presence of two Lin^−^ CD123^+^ populations in PB and suggest that CD123^+^ CD127^low^ cells express a mixture of basophil and lymphoid features.

### A Human Lin^−^ CD123^+^ CD127^low^ CLA^+^ Population with ILC Features in PB

**Figure 2:**
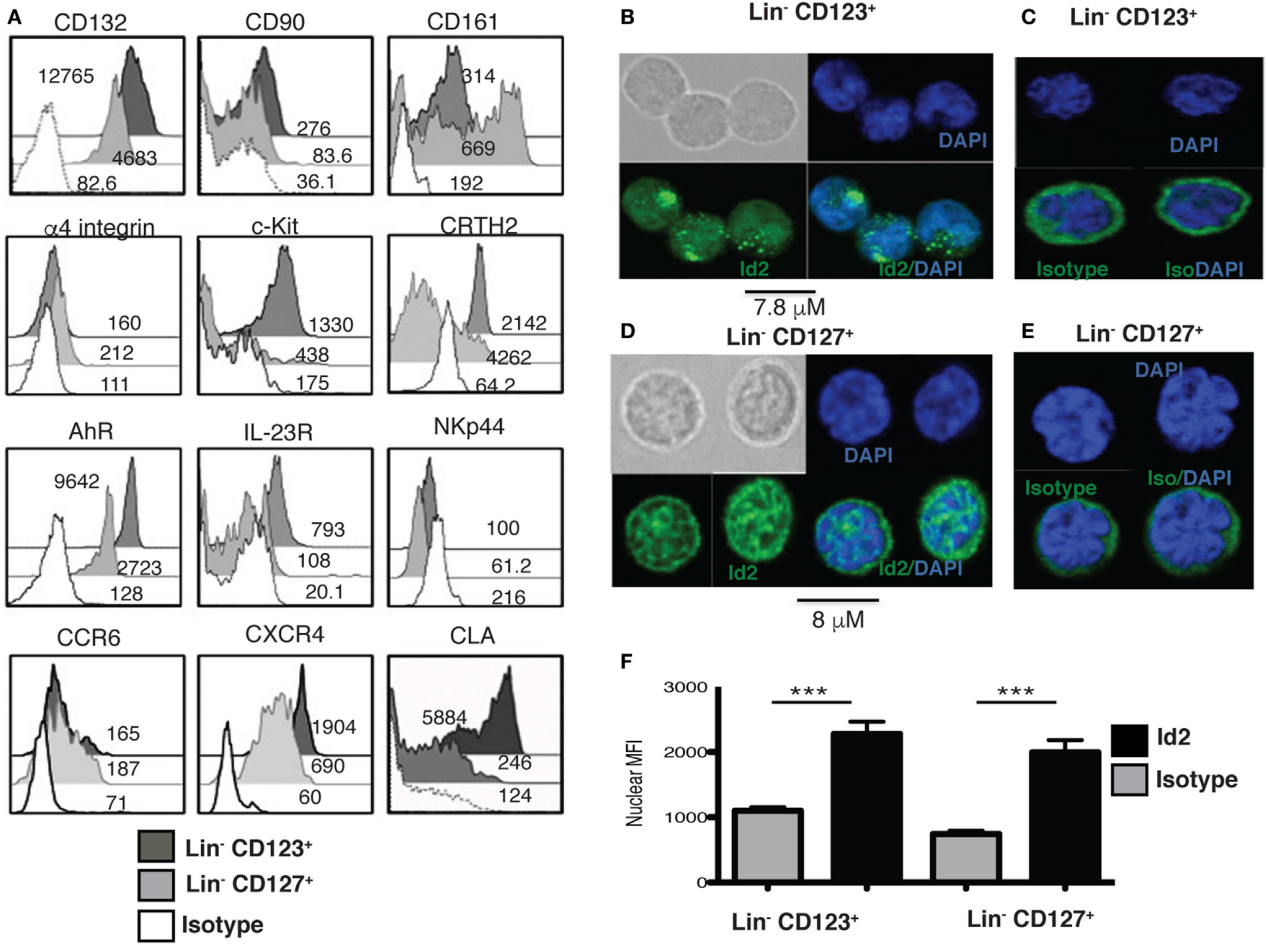
**A human Lin^−^ CD123^+^ CD127^low^ CLA^+^ population with innate lymphoid cells (ILC) features in peripheral blood**. **(A)** Compared expression of multiple ILC markers in the Lin^−^ CD123^+^ CD127^low^ and Lin^−^ CD127^+^ populations: black: Lin^−^ CD123^+^, gray: Lin^−^ CD127^+^, and white: isotype. Median fluorescence intensity values representative of three independent experiments are shown. **(B)** Inhibitor of DNA binding 2 (Id2) expression in sorted Lin^−^ CD123^+^ from the lymphoid region (gated as shown in Figure S1A in Supplementary Material) **(C)** isotype and **(D)** Lin^−^ CD127^+^ cells, **(E)** isotype, immunofluorescence staining for Id2 (green) and DAPI (nucleus staining), *n* = 10. **(F)** Nuclear median fluorescence intensity in Lin^−^ CD123^+^ and Lin^−^ CD127^+^ compared with the isotype control (***p < 0.0001).

Several ILC markers were investigated in the Lin^−^ CD123^+^ CD127^low^ within the lymphoid region. Importantly, such population expressed high levels of CD132 and CD90, compared to the Lin^−^ CD127^+^ population, but low levels of CD161 (Figure 2A). In addition, both populations were similar in the low expression of α4 integrin that has been reported in ILC precursors. Higher levels of c-Kit and CRTH2, AhR and IL23R were observed in the Lin^−^ CD123^+^ CD127^low^ when compared to the Lin^−^ CD127^+^ counterpart, but both populations were NKp44^−^. Although both populations displayed similar amounts of CCR6, the Lin^−^ CD123^+^ CD127^low^ population exhibited a remarkable expression of CXCR4 and of the CLA (Figure 2A).

In addition, the transcriptional factor Id2, which is crucial for ILC commitment, was confirmed in these cells. Notably, the Lin^−^ CD123^+^ CD127^low^ and CD127^+^ populations express Id2 predominantly within nuclei compared to the isotype control, although the staining pattern was distinct (Figures 2B–F).

### Identity and Maintenance of the ILC Features in the Lin^−^ CD123^+^ CD127^low^ Population Are Mediated by IL-3

**Figure 3:**
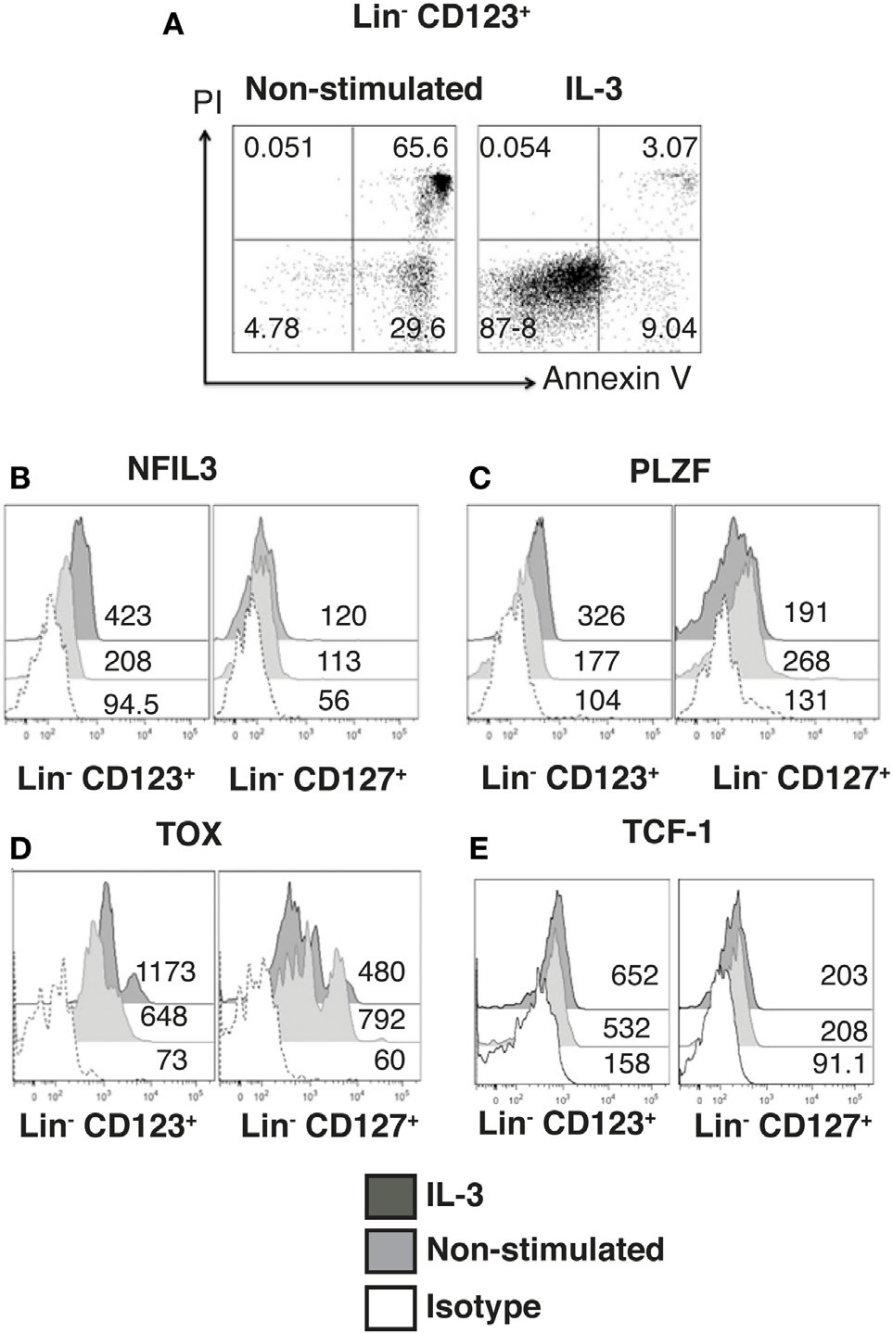
**Identity and maintenance of the innate lymphoid cell features in the Lin^−^ CD123^+^ CD127^low^ population are mediated by IL-3**. **(A)** Viability assay with sorted Lin^−^ CD123^+^ cells (from the lymphoid region, gated as shown in Figure S1A in Supplementary Material) cultured ± IL-3 for 3 days. The percentages of dead and viable cells are shown. Dot plots are representative of three independent experiments. **(B–E)** Comparison of NFIL3, promyelocytic leukemia zinc finger (PLZF), thymocyte selection-associated high-mobility group box protein (TOX), and TCF-1 expression in Lin^−^ CD123^+^ and Lin^−^ CD127^+^ populations (from total peripheral blood mononuclear cells): black: +IL-3 (18 h), gray: non-stimulated, and dotted: isotype. Median fluorescence intensity values representative of three experiments are shown.

Next, the effect of IL-3 was assessed in the Lin^−^ CD123^+^ CD127^low^ population. Sorted Lin^−^ CD123^+^ CD127^low^ from the lymphoid region cells were cultured in the presence of IL-3; after 3 days, IL-3 enabled the survival of the cells, as more than 90% of the cells cultured without IL-3 died via apoptosis (Figure 3A). In addition to Id2 NFIL3, PLZF, TOX, and TCF-1 have been reported as crucial in ILC development (15–17). Notably, the Lin^−^ CD123^+^ CD127^low^ population expressed similar levels of NFIL3 compared with the classic CD127^+^ ILC; however, the CD127^+^ population exhibited a higher expression of PLZF, TOX, and TCF-1. Remarkably, IL-3 was capable of increasing the expression of NFIL3, PLZF, and TOX in the Lin^−^ CD123^+^ CD127^low^ population in contrast to the CD127^+^ population (Figures 3B–E). These findings suggest that IL-3 has two main roles in the CD123^+^ CD127^low^ population: maintenance of cell survival and the upregulation of crucial transcriptional factors related to ILC identity.

### The Lin^−^ CD123^+^ CD127^low^ Population Is Primarily a Conspicuous Population That Expresses T-bet and RORγt

**Figure 4:**
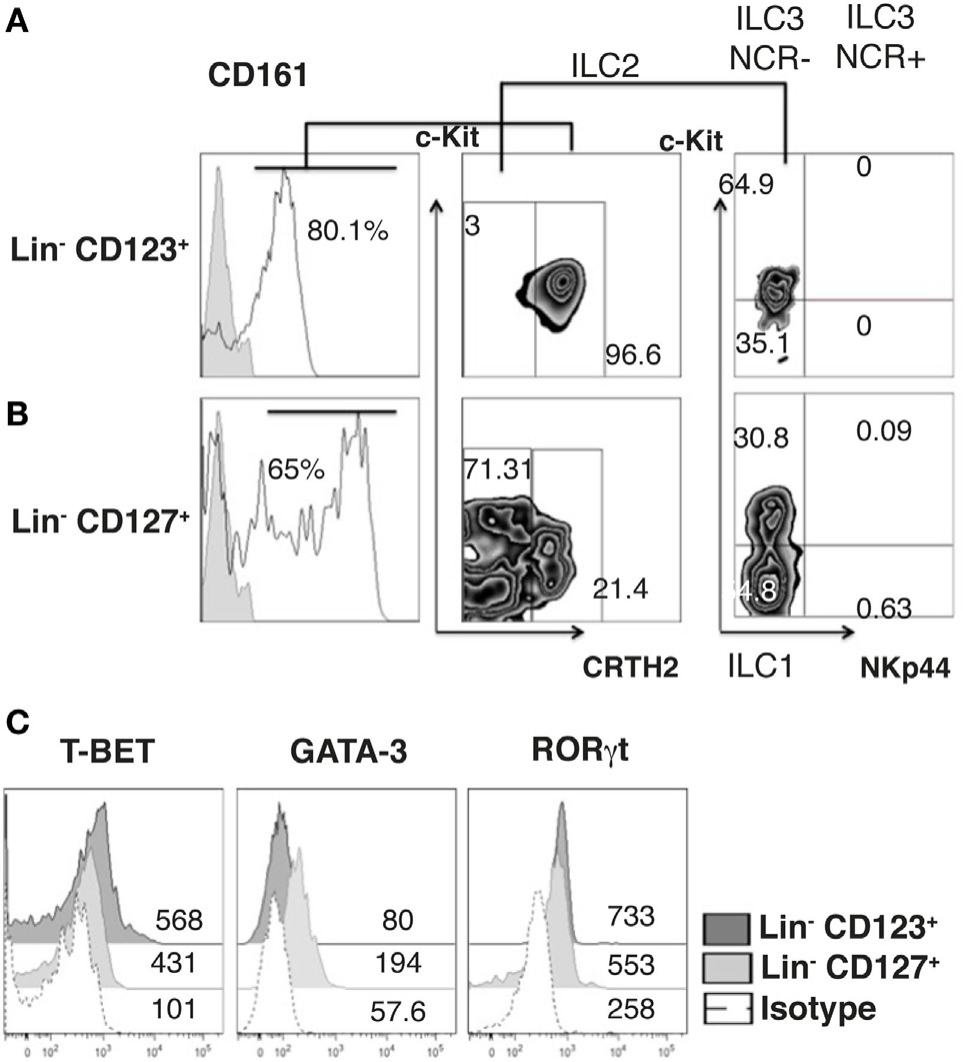
**The Lin^−^ CD123^+^ CD127^low^ population is primarily a conspicuous population that expresses T-bet and RORγt**. **(A)** Classification of innate lymphoid cell (ILC) subpopulations (from total peripheral blood mononuclear cells): gated on: CD161^+^ cells; ILC1 (CRTH2^−^, c-Kit^−^), ILC2 (CRTH2^+^), and ILC3 (CRTH2^−^, c-Kit^+^) subpopulations within the Lin^−^ CD123^+^ **(A)** and Lin^−^ CD127^+^ **(B)** populations. Dot plots are representative of at least three independent experiments. **(C)** T-bet, GATA-3, and ROR-γt expression on Lin^−^ CD123^+^ and Lin^−^ CD127^+^ populations: black: Lin^−^ CD123^+^, gray: Lin^−^ CD127^+^, and dotted: isotype. Median fluorescence intensity values representative of three independent experiments are presented.

Innate lymphoid cells populations are classified by phenotype in ILC1, ILC2, and ILC3 (44). In this regard, we determined that the majority of cells within the Lin^−^ CD123^+^ CD127^low^ population displayed a phenotype similar to ILC2, due to expression of CD161^low^, c-Kit, and CRTH2, although expression of CRTH2 may resemble a basophil-like phenotype. In contrast, a limited number of cells with an ILC1 (CRTH2^−^, c-Kit^−^) or ILC3 (CRTH2^−^, c-Kit^+^, NKp44^−^) phenotype were identified (Figure 4A). In contrast, in the CD127^+^ ILC, we identified two populations by the expression of CD161, and ILC1, ILC2, and ILC3 (NKp44) populations were identified (Figure 4B). Remarkably, the Lin^−^ CD123^+^ CD127^low^ population had similar expressions of T-bet and RORγt compared with the CD127^+^ population; however, there was only a slight expression of GATA-3 (Figure 4C). The small amount of diversity and the expression of T-bet, RORγt, or GATA were not affected in the Lin^−^ CD123^+^ CD127^low^ population by IL-3 culture (data not shown). These findings indicated that the Lin^−^ CD123^+^ CD127^low^ population in PB is primarily a conspicuous population with minority phenotypic diversity that expresses T-bet and RORγt, which suggests that these transcription factors may be involved in their function.

### Steady-State Peripheral Lin^−^ CD123^+^ CD127^low^ Population Expresses Limited Th Type Cytokine Variety

**Figure 5:**
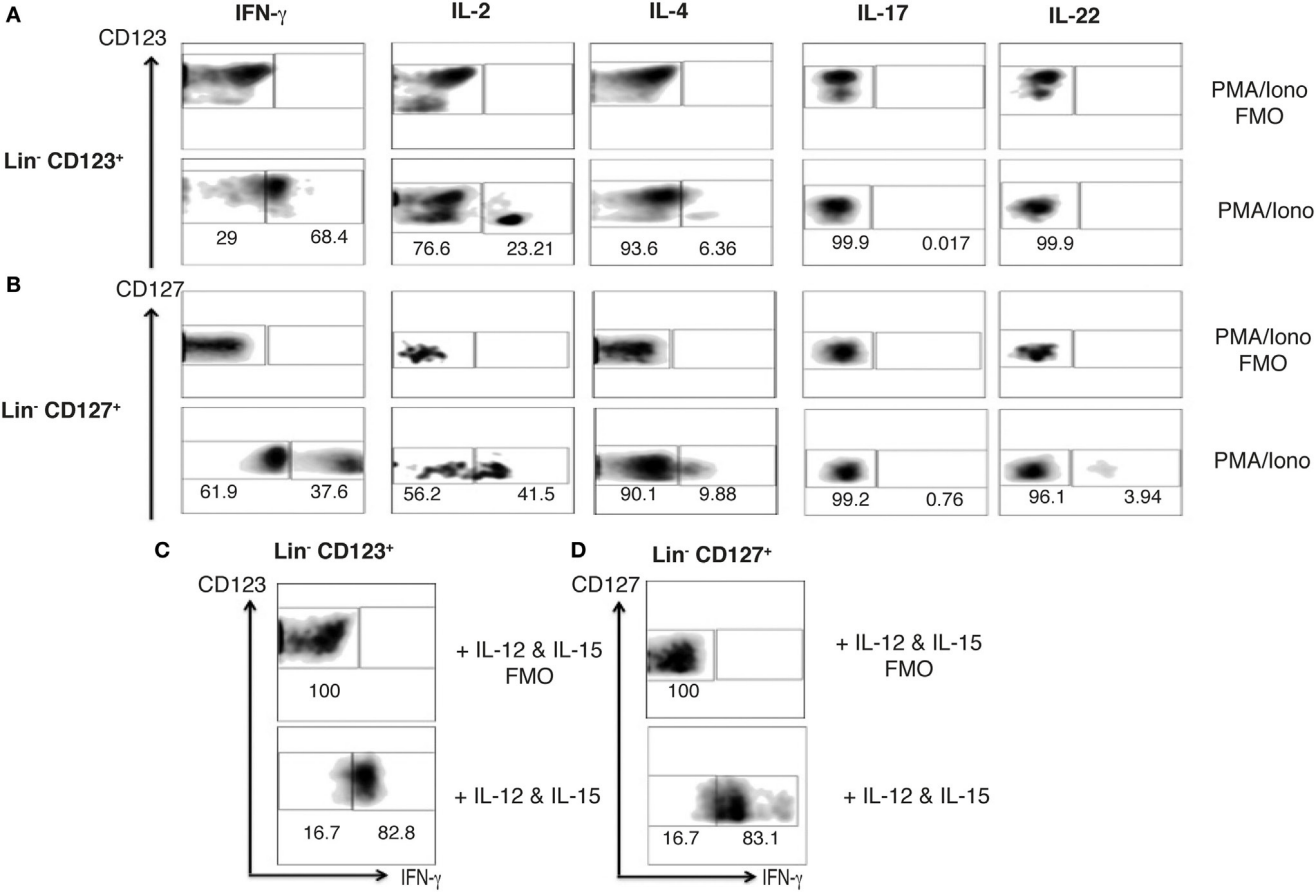
**Steady-state peripheral Lin^−^ CD123^+^ CD127^+^ population expresses limited T helper type cytokine variety**. Percentages of positive and negative cells that express IFN-γ, IL-2, and IL-4; IL-17 and IL-22 in the presence of PMA/Iono (6 h) **(A)** Lin^−^ CD123^+^ and Lin^−^ CD127^+^ **(B)** populations within peripheral blood mononuclear cells. **(C,D)** IFN-γ expression in Lin^−^ CD123^+^ and Lin^−^ CD127 after 18 h of activation with the ILC1 cocktail (IL-12 + IL-15). In each panel, FMO controls are shown in the upper panels. Density plots are representative of at least three independent experiments. FMO, fluorescence minus one control.

The ILC1, 2, and 3 populations have been described as innate analogs to Th lymphocytes by their capacity to express cytokines (28, 45). Therefore, to determine the Th type cytokine production of the Lin^−^ CD123^+^ CD127^low^ population, the expression of intracellular cytokines in activated PBMCs was assessed. The Lin^−^ CD123^+^ CD127^low^ population expresses IFN-γ, IL-2, and IL-4 after PMA/Ionomycin activation (Figure 5A). Of note, the population that mainly expresses the cytokines downregulates CD123. The cytokine expression pattern was similar in the classic CD127^+^ ILC population (Figure 5B). Remarkably, the expression of IL-17 or IL-22 was not identified in the PB Lin^−^ CD123^+^ CD127^low^ population, and only a minor percentage of IL-22^+^ in the CD127^+^ ILC was identified. Considering that it has been reported that ILC may be activated by cytokines, PBMCs were cultured with cytokine cocktails for ILC1, ILC2, or ILC3 activation. Figures 5C,D indicate that IFN-γ is expressed in the Lin^−^ CD123^+^ CD127^low^ and CD127^+^ populations after culture with IL-12 and IL-15. The percentage of IFN-γ^+^ cells is similar in both populations; however, the expression of IFN-γ is increased in the CD127^+^ compared with the Lin^−^ CD123^+^ CD127^low^ ILC population. The culture with the ILC2 cocktail (IL-33 and IL-2) did not induce the expression of ILC2 type cytokines, such as IL-4 or IL-13, and the culture with the ILC3 cocktail (IL-1β, IL-2, and IL-23) did not induce the expression of ILC3 type cytokines, such as IL-17 or IL-22 (Figure S3 in Supplementary Material). These findings indicate that the steady-state peripheral Lin^−^ CD123^+^ CD127^low^ population expresses limited Th type cytokine variety.

### The Lin^−^ CD123^+^ CD127^low^ Population in PB Expresses High Levels of IL-8

**Figure 6:**
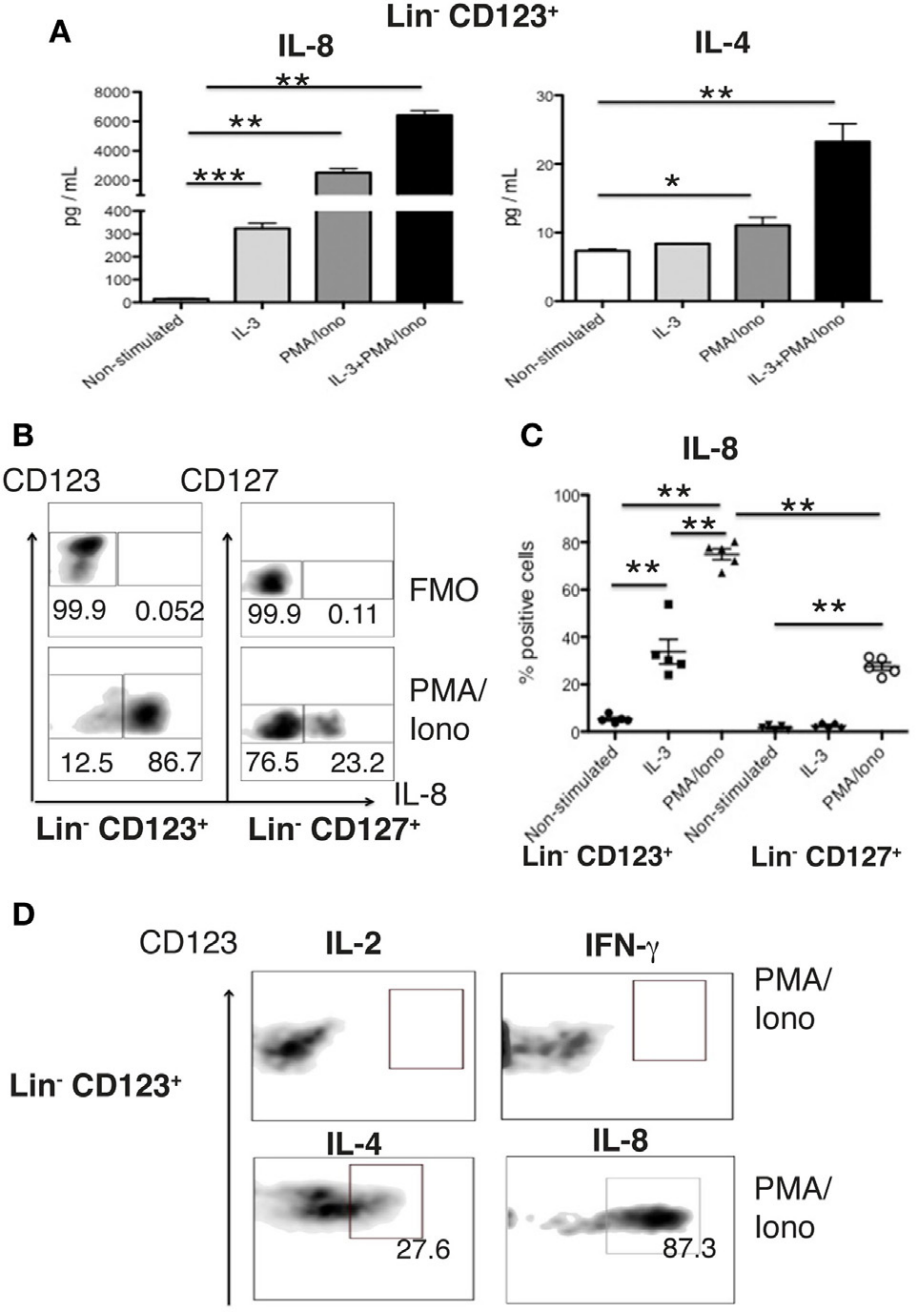
**The Lin^−^ CD123^+^ CD127^low^ population in PB expresses high levels of IL-8**. **(A)** IL-8 and IL-4 quantification in culture supernatants from isolated Lin^−^ CD123^+^ cells (within the lymphoid region gated as depicted in Figure S1A in Supplementary Material) cultured for 18 h ± IL-3, PMA/Iono or IL-3 + PMA/Iono. **(B)** Percentages of positive and negative cells that express IL-8 (lower panel) in the presence of PMA/Iono (6 h) in Lin^−^ CD123^+^ and Lin^−^ CD127^+^ populations within peripheral blood mononuclear cells (PBMCs). Upper panel: FMO control for each cell population. Dot plots are representative of at least three independent experiments. **(C)** Percentage of Lin^−^ CD123^+^ and Lin^−^ CD127^+^ IL-8^+^ cells within PBMCs ± IL-3 and PMA/Iono (*n* = 5). **(D)** Expression of IFN-γ, IL-2, IL-4, and IL-8 in sorted (as in Figure 1) in Lin^−^ CD123^+^ cultured in the presence of PMA/Iono for 6 h. Density plots are representative of at least three independent experiments. FMO, fluorescence minus one control (**p < 0.01).

We subsequently isolated the Lin^−^ CD123^+^ CD127^low^ population from the lymphoid region and CD127^+^ ILC population to evaluate the cytokines in the supernatants via a multiplex assay after activation. Surprisingly, the Lin^−^ CD123^+^ CD127^low^ population produced high levels of IL-8 and low levels of IL-4 (Figure 6A), whereas the CD127^+^ expressed IL-8 and IL-2; however, other Th type cytokines were not identified in the purified populations (data not shown). The high expression of IL-8 by the CD123^+^ CD127^low^ population was confirmed via intracellular detection in which IL-3 induced the expression of IL-8 (Figure 6C), which was significantly increased in the presence of PMA/Ionomycin (Figures 6B,C). The percentage of IL-8^+^ cells was significantly increased in the Lin^−^ CD123^+^ CD127^low^ cells compared with the CD127^+^ ILC (Figures 6B,C). The lack of expression of IL-2 and IFN-γ in the isolated Lin^−^ CD123^+^ CD127^low^ population was also confirmed via intracellular detection, in which only the expressions of IL-4 and IL-8 (Figure 6D, lower panel) and not IL-2 or IFN-γ were identified (Figure 6D, upper panel). These findings indicate that the freshly isolated steady-state Lin^−^ CD123^+^ CD127^low^ population expresses high levels of IL-8 and confirm the limited expression of Th type cytokines.

### The Lin^−^ CD123^+^ CD127^low^ CLA^+^ Population Has Migratory Potential Mediated by SDF-1

**Figure 7:**
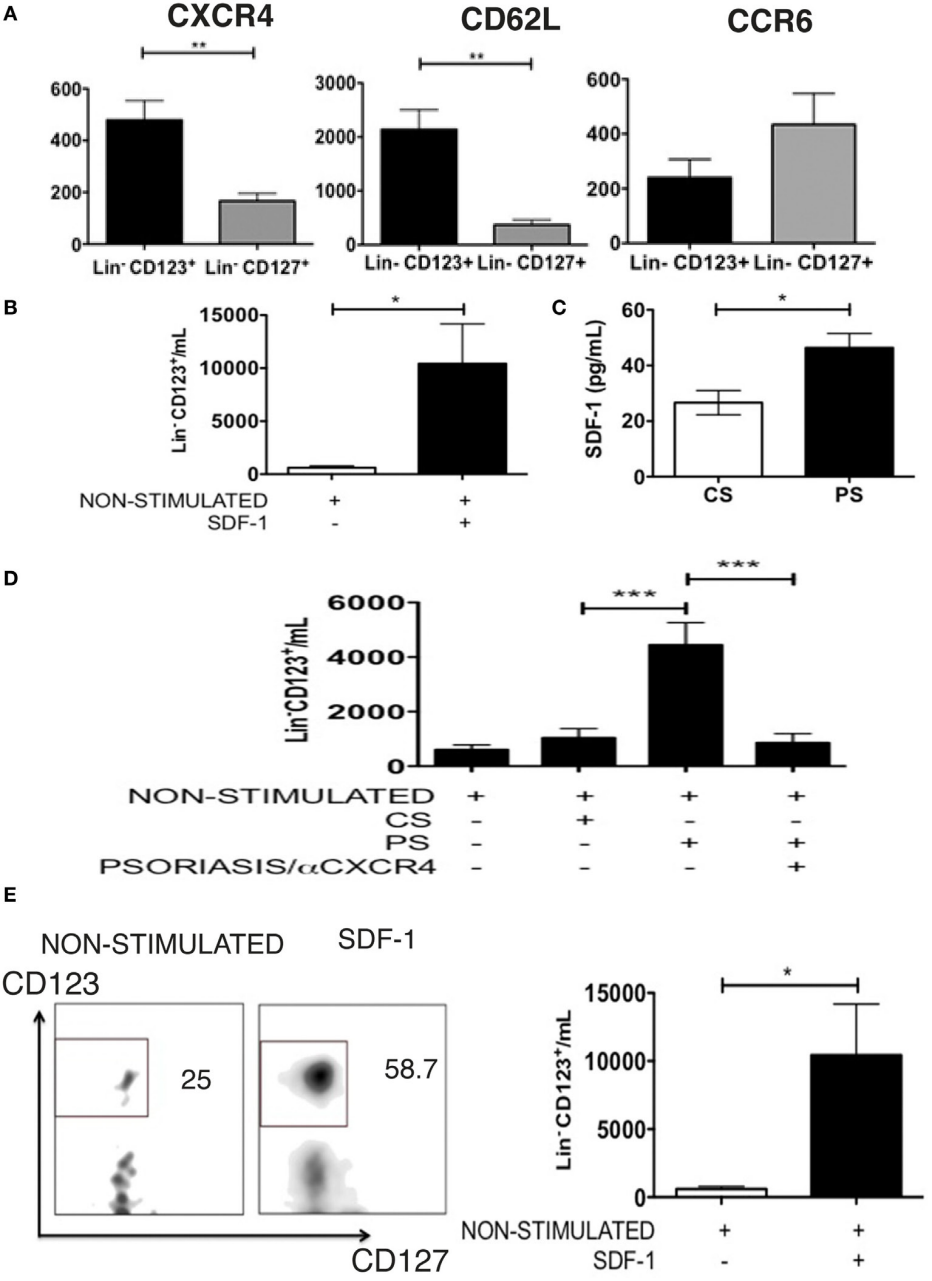
**The Lin^−^ CD123^+^ CD127^low^ CLA^+^ population has migratory potential mediated by SDF-1**. **(A)** CXCR4 (*N* = 6), CD62L (*N* = 5), and CCR6 (*N* = 6) expression in Lin^−^ CD123^+^ and Lin^−^ CD127^+^ cells from PB of healthy donors; bar graph representation of median fluorescence intensity values (*n* = 6). **(B)** Migration assay of Lin^−^ CD123^+^ [from total peripheral blood mononuclear cells (PBMCs)] in the presence of SDF-1. **(C)** Amount of SDF-1, quantified by ELISA, in supernatants from control skin (CS) or psoriasis skin (PS) cell cultures. **(D)** Migration assay of Lin^−^ CD123^+^ cells (from total PBMCs) in the presence of supernatants from CS or PS cell cultures ± blocking anti-CXCR4. **(E)** Transmigration assay of Lin^−^ CD123^+^ cells (from total PBMCs) in the presence of activated endothelial cells and SDF-1 (*N* = 10). Dot plots on the left indicate the percentage of Lin^−^ CD123^+^ migrating cells and are representative of at least three independent experiments (*p < 0.05, **p < 0.01, ***p < 0.001).

Considering the high display of CLA in the Lin^−^ CD123^+^ CD127^low^ population, the expression of other homing receptors was subsequently assessed. Figure 7A indicates that the Lin^−^ CD123^+^ CD127^low^ population exhibited an increased expression of CXCR4 and CD62L compared with the CD127^+^ ILC. In contrast, we identified an increased expression of CCR6 in the CD127^+^ ILC compared with the Lin^−^ CD123^+^ CD127^low^ cells (Figure 7A). The Lin^−^ CD123^+^ CD127^low^ population from PB highly expresses CXCR4; thus, migration assays were performed with SDF-1, the ligand for CXCR4. As indicated in Figure 7B, the Lin^−^ CD123^+^ CD127^low^ population from PB migrates in response to SDF-1. In addition, SDF-1 is present in the skin culture supernatants from CS, and a significant increase in SDF-1 was identified in the inflamed skin cultures obtained from the biopsies of patients with psoriasis (Figure 7C). Remarkably, a significantly increased migration in response to supernatants obtained from cell cultures from PS lesion biopsies was dependent on CXCR4-SDF-1 compared with supernatants from the CS (Figure 7D). In addition, the Lin^−^ CD123^+^ CD127^low^ but not the CD127^+^ population transmigrated through activated endothelial, and this migration significantly increased in response to SDF-1 (Figure 7E). Interestingly, the Lin^−^ CD123^+^ CD127^low^ population increased the expression of CD127 after contact with activated endothelial cells (Figure S5 in Supplementary Material). These findings indicate the high migratory capability of the Lin^−^ CD123^+^ CD127^low^ CLA^+^ population and suggest that under inflammatory conditions, SDF-1 could promote skin infiltration of Lin^−^ CD123^+^ CD127^low^ cells.

### The Lin^−^ CD123^+^ CD127^low^ Population within the Lymphoid Region Downregulates CD123 and Basophil Markers upon Activation

**Figure 8:**
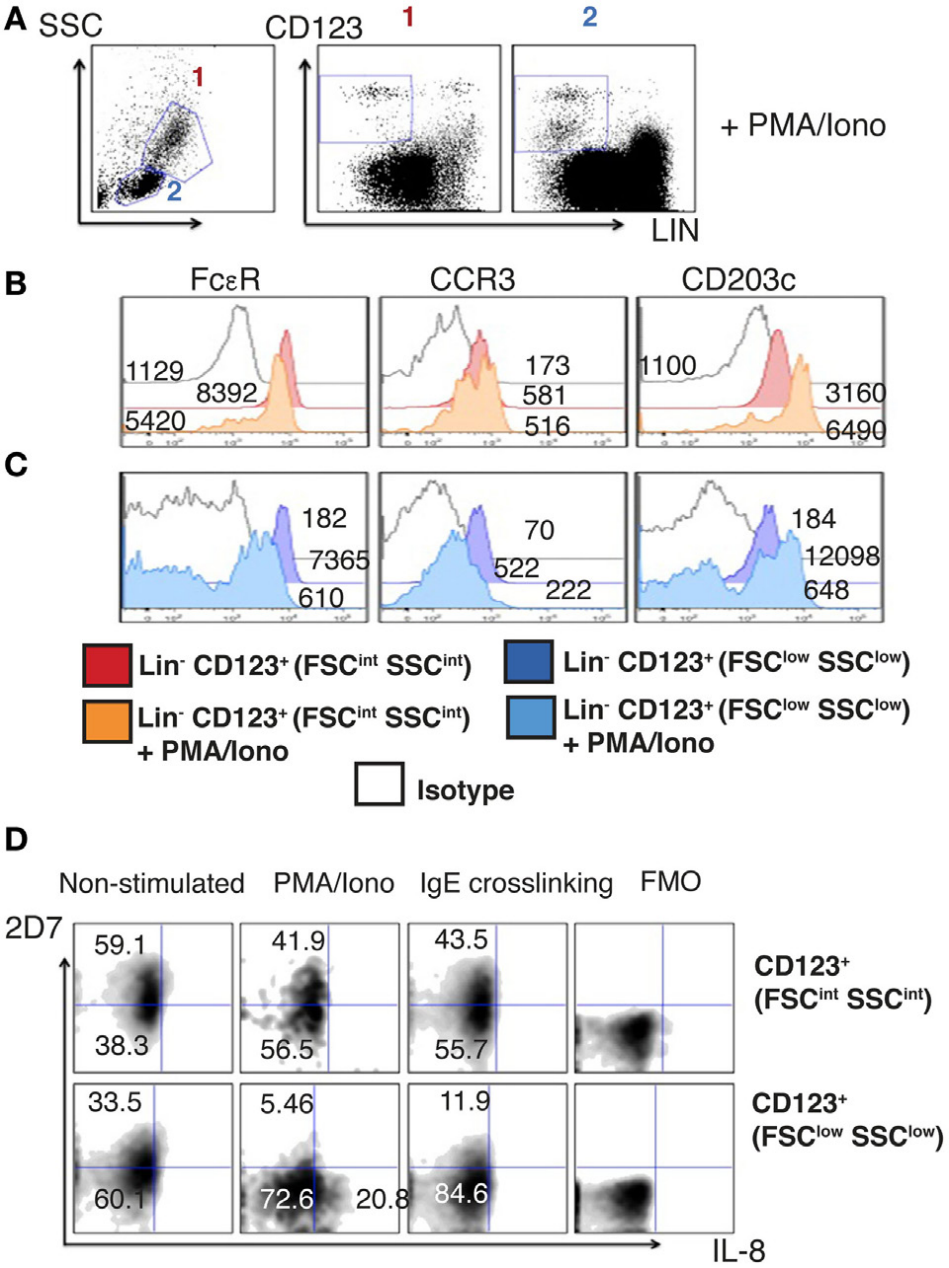
**The Lin^−^ CD123^+^ CD127^low^ population from the lymphoid region after activation downregulates CD123 and basophil markers**. **(A)** Prospective identification of two CD123^+^ cell populations in the FSC^int^ SSC^int^ and FSC^low^ SSC^low^ fractions as FSC^int^ SSC^int^ (1) and FSC^low^ SSC^low^ (2) after activation with PMA/Iono (6 h). **(B,C)** Basophil markers expression after PMA/Iono (6 h) stimulation in the Lin^−^ CD123^+^ (FSC^int^ SSC^int^) and Lin^−^ CD123^+^ (FSC^low^ SSC^low^). Red: non-stimulated and orange: stimulated Lin^−^ CD123^+^ (1: FSC^int^ SSC^int^). Dark blue: non-stimulated and light blue: stimulated Lin^−^ CD123^+^ (2: FSC^low^ SSC^low^). White: isotype. Median fluorescence intensity values representative of three independent experiments are presented. **(D)** 2D7 and IL-8 expression after PMA/Iono (6 h) stimulation or IgE crosslinking. Upper panel: Lin^−^ CD123^+^ (FSC^int^ SSC^int^) and lower panel: Lin^−^ CD123^+^ (FSC^low^ SSC^low^). FMO: for each population is shown at the right of each panel. Density plots are representative of at least three independent experiments. FMO, fluorescence minus one control.

The majority of Lin^−^ CD123^+^ CD127^low^ cells within the lymphoid region downregulates CD123 and basophil markers such as FcεR, CCR3, and CD203c upon activation. In contrast, most CD123^+^ FSC^int^ SSC^int^ counterparts maintain the expression of these molecules (Figures 8A–C).

Considering that a reduction of the antigen recognized by 2D7 has been reported in activated basophils, the expression of IL-8 and 2D7 was evaluated after activation with PMA/Ionomycin and by IgE crosslinking. Remarkably, the population expressing IL-8 is the CD123^low^ and not the one maintaining high expression of CD123. We did not observe IL-8 expression after IgE crosslinking. In contrast, both cell populations reduced the expression of 2D7 in response to PMA/Ionomycin and IgE (Figure 8D). These results clearly show important functional differences between the lymphoid Lin^−^ CD123^+^ CD127^low^ population and the CD123^+^ FSC^int^ SSC^int^ one, suggesting that the Lin^−^ CD123^+^ CD127^low^ population from the lymphoid region may require activation to acquire ILC functional features and to diminish basophil activity.

### Barrier Tissues, Such As Skin, Are Normally Infiltrated by a Specialized Lin^−^ CD123^low^ CD127^int^ Population with ILC Features

**Figure 9:**
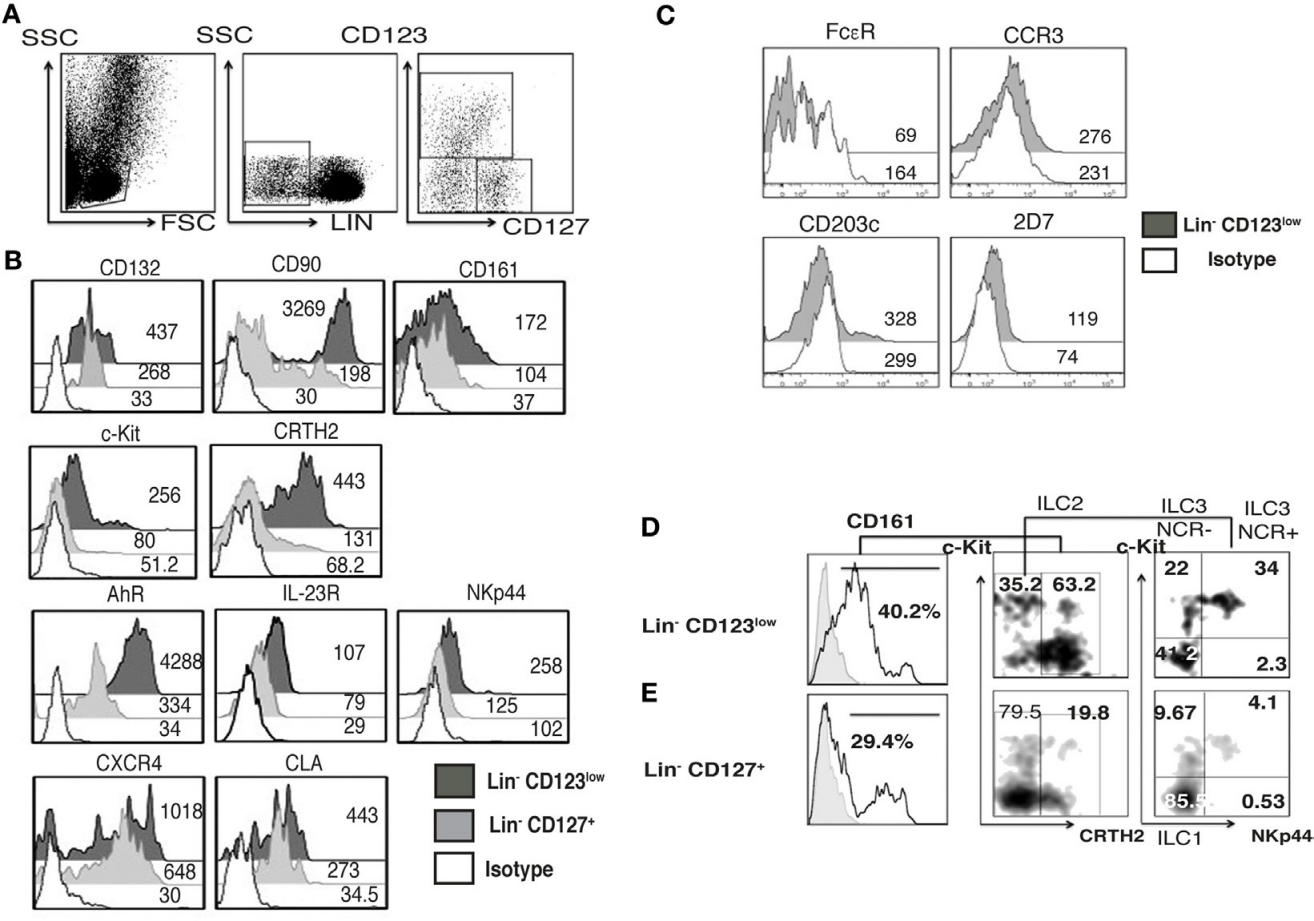
**Barrier tissues, such as skin, are normally infiltrated by specialized Lin^−^ CD123^low^ CD127^int^ with innate lymphoid cell (ILC) features**. Total skin cells from the epidermis of control skin (CS) were analyzed as in peripheral blood. **(A)** From left to right: gate analysis on lymphoid cells, exclusion of lineage positive cells (CD3^+^, CD14^+^, CD19^+^, CD94^+^, HLA-DR^+^), and identification of Lin- cells: Lin^−^ CD123^low^ and Lin^−^ CD127^+^ populations. **(B)** Compared expression of multiple ILC markers in the Lin^−^ CD123^low^ CD127^+^ and Lin^−^ CD127^+^ populations. **(C)** Basophil markers expression in the Lin^−^ CD123^low^ from CS. Black: Lin^−^ CD123^low^, gray: Lin^−^ CD127^+^, and white: isotype. Median fluorescence intensity values representative of three independent experiments are presented. **(D,E)** Classification of skin ILC subpopulations: gated on: CD161^+^ cells; ILC1 (CRTH2^−^, c-Kit^−^), ILC2 (CRTH2^+^), and ILC3 (CRTH2^−^, c-Kit^+^) subpopulations within the Lin^−^ CD123^low^ **(D)** and Lin^−^ CD127^+^ **(E)** populations. Density plots and histograms are representative of at least three independent experiments.

**Figure 10:**
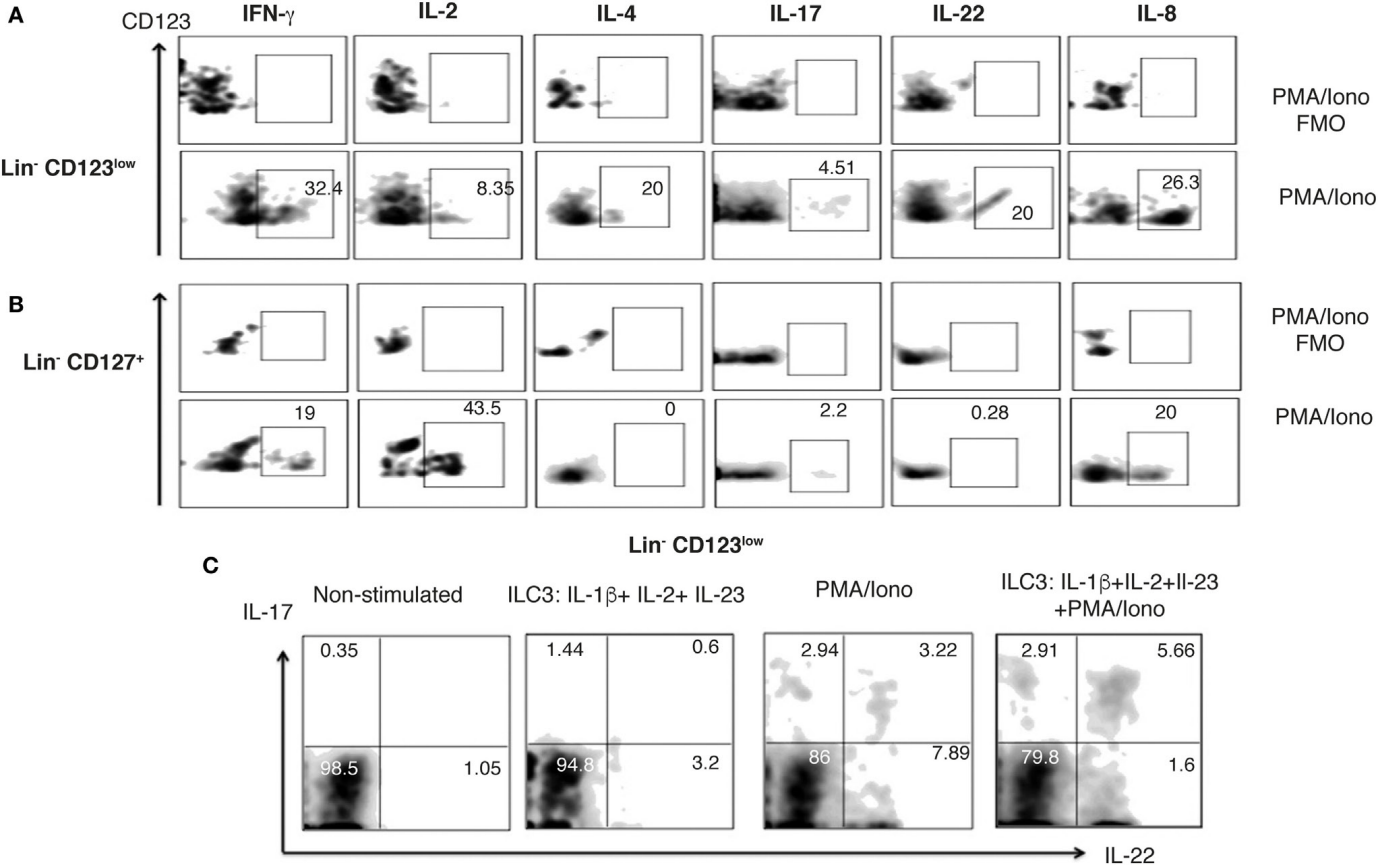
**Skin Lin^−^ CD123^**low**^ cells produce IL-17 and IL-22**. **(A,B)** Percentages of positive and negative total skin cells that express IFN-γ, IL-2, IL-4; IL-17, IL-22, and IL-8 in the presence of PMA/Iono (6 h) in the Lin^−^ CD123^low^ **(A)** and Lin^−^ CD127^+^ **(B)** populations within total control skin cells. Upper panels: FMO controls. **(C)** Expression of IL-17 and IL-22 in the Lin^−^ CD123^low^ skin cells cultured for 18 h in the presence of IL-1β, IL-2, and IL-23 (ILC3 activation cocktail) ± PMA/Iono (last 6 h) or ILC3 activation cocktail + PMA/Iono (last 6 h). Density plots are representative of at least three independent experiments. FMO, fluorescence minus one control.

Considering the high expression of CLA in the Lin^−^ CD123^+^ CD127^low^ population and the downregulation of CD123 after activation next, we investigated whether an equivalent CD123^+^ population might be identified in the skin. We found Lin^−^ CD123^low^ and Lin^−^ CD127^+^ cells in the dermis of CS (Figure 9A). Notably, in the skin, the Lin^−^ CD123^low^ population exhibited an increased expression of CD127 and CD90 compared with the PB CD123^+^ CD127^low^ population; however, it expressed lower levels of CD132 and CD161. The Lin^−^ CD123^low^ population in skin is positive for c-Kit, CRTH2, AhR, and IL-23 and expresses high levels of NKp44 compared with the CD127^+^ population. Although similar levels of CLA and CXCR4 are recorded (Figure 9B), the Lin^−^ CD123^low^ population does not express basophil markers such as FcεR, CCR3, CD203c, or 2D7 (Figure 9C). Surprisingly, among the Lin^−^ CD123^low^ population in the CS, ILC1, ILC2, and ILC3 (NKP44^+^ and NKP44^−^) may be identified by phenotype (Figure 9D). Among the CD127^+^ ILC population, ILC1, ILC2, and ILC3 (NKp44^+^ and NKp44^−^) were also identified (Figure 9E). However, the proportions are different compared with the Lin^−^ CD123^low^ skin population and the PB CD127^+^ ILC, as well as between different CS samples (data not shown). Considering that the Th cytokine production by ILC has mainly been reported in peripheral tissues, the cytokine production by the CS Lin^−^ CD123^low^ population was subsequently evaluated. After activation, the Lin^−^ CD123^low^ population present in CS expresses IFN-γ, IL-2, and IL-4 and, remarkably, IL-17 and IL-22 (Figure 10A). The CD127^+^ ILC population exhibited a similar pattern of cytokine expression; however, the percentages were different between both populations (Figure 10B) and between different CS biopsies. As a result of the expression of IL-17 and IL-22 after PMA/Ionomycin activation, we subsequently assessed whether the Lin^−^ CD123^low^ population responded to the ILC3 cocktail (IL-1β, IL-2, and IL-23) in CS. CS was stimulated with the ILC3 cocktail, PMA/Ionomycin or the combination. Figure 10C indicates that the Lin^−^ CD123^low^ population exhibits a minor expression of IL-22 or IL-17 in response to the ILC3 cocktail; however, increases in the percentages of IL-22^+^, IL-17^+^, and both were identified in response to the combination of ILC3 cocktail and PMA/Ionomycin compared with only PMA/Ionomycin. The expression of IL-17 by the skin Lin^−^ CD123^low^ cells and the CD127^+^ populations correlates with the expression of RORγt (Figure S6 in Supplementary Material) and with the ILC3 subset phenotype. Regarding the IL-8 expression, we determined that after stimulation, the Lin^−^ CD123^low^ population from CS expressed IL-8 to a lesser extent compared with the PB Lin^−^ CD123^+^ CD127^low^ population (Figure 10A). These findings indicate that in accordance with the high expression of CLA in the Lin^−^ CD123^+^ CD127^low^ population from PB and with the downregulation of CD123 after activation, we identified a Lin^−^ CD123^low^ population in CS that exhibit increased phenotypic and Th type cytokine diversity compared with its PB counterpart. These findings strongly suggest that specialized Lin^−^ CD123^low^ CD127^int^ population with ILC features normally infiltrate barrier tissues, such as skin, which appear to be in a late stage of activation.

### SDF-1 Dependent Migration of Lin^−^ CD123^+^ CD127^low^ May Precede Activation and Local Production of IL-17 and IL-22 in Psoriasis Patients

**Figure 11:**
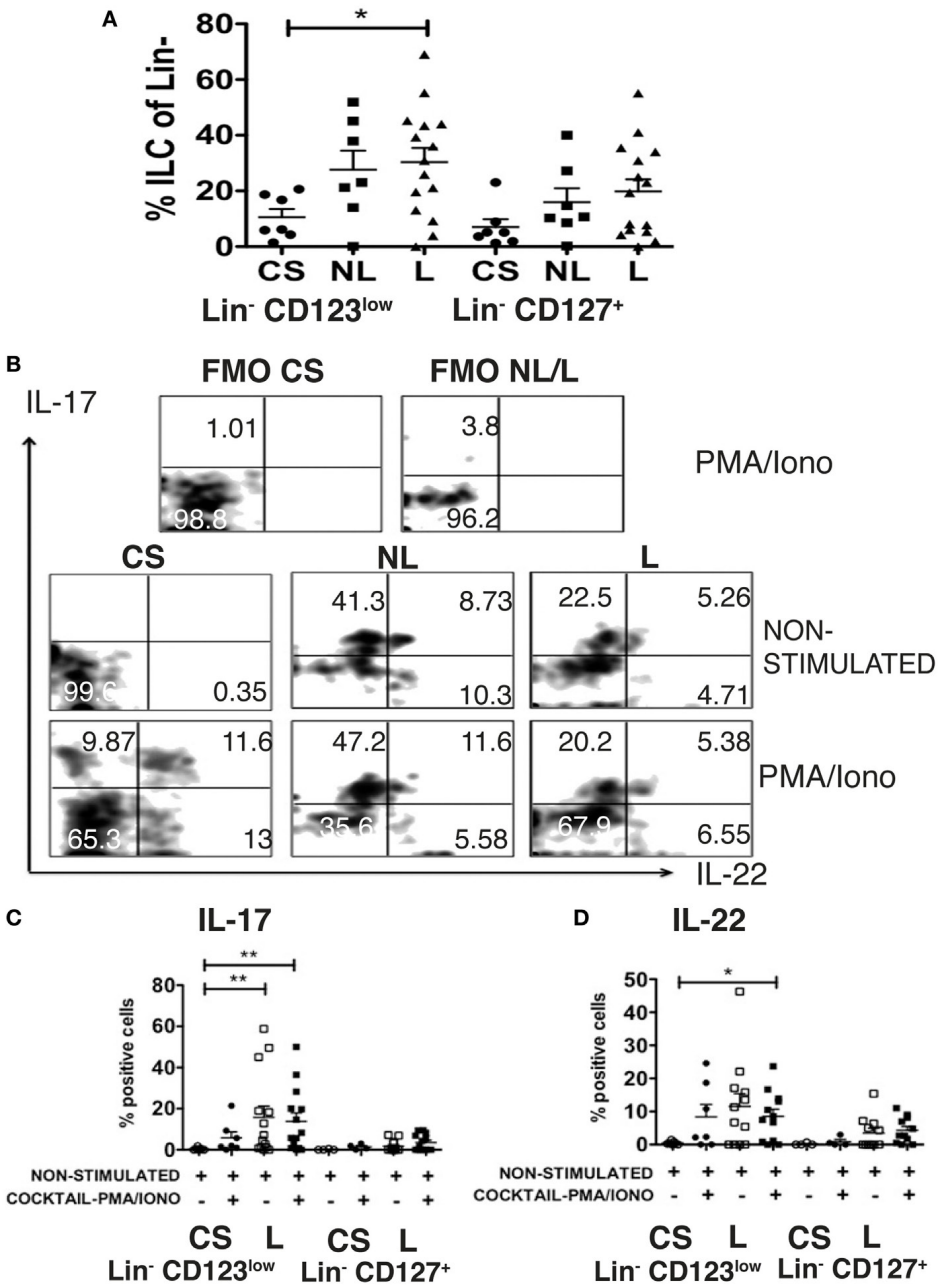
**SDF-1-dependent migration of Lin^−^ CD123^low^ CD127^low^ cells may precede activation and local production of IL-17 and IL-22 in psoriasis patients**. **(A)** Frequencies of Lin^−^ CD123^low^ and Lin^−^ CD127^+^ populations in control skin (CS) *N* = 7, non-lesioned (NL) skin *N* = 7, and lesioned (L) *N* = 15 skin from psoriasis patients. **(B)** Intracellular expression of IL-17 and IL-22 in Lin^−^ CD123^low^ cells from CS, NL and L skin cultured during 18 h ± IL-1β, IL-2, and IL-23 (ILC3 activation cocktail) ± PMA/Iono (last 6 h) Dot plots are representative of at least three independent experiments. FMO, fluorescence minus one control. **(C,D)** Percentages of skin IL-17^+^ and IL-22^+^ Lin^−^ CD123^low^ cells (CS: *N* = 7 and L: *N* = 12) or Lin^−^ CD127^+^ cells (CS: *N* = 4 and L: *N* = 11) cultured during 18 h ± IL-2, IL-23, and IL-1β (ILC3 activation cocktail) + PMA/Iono (*p < 0.05, **p < 0.01).

As a result of the migratory potential and the ability of the Lin^−^ CD123^low^ CD127^int^ population present in CS to express IL-17 and IL-22, the frequencies and the expression of these cytokines by the ILC-related populations from psoriasis patients were subsequently assessed. Importantly, a significant increase in the frequencies of the Lin^−^ CD123^low^ CD127^int^ population was identified in the skin lesions of psoriasis patients compared with the CS. Interestingly, an increase was also identified in the non-lesioned (NL) skin of psoriasis patients. We also identified an increase in the CD127^+^ population in the NL and lesioned skin from psoriasis patients (Figure 11A). The Lin^−^ CD123^low^ population in the CS expressed IL-17 and IL-22 only after activation. However, in some patients, the Lin^−^ CD123^low^ CD127^int^ population from the lesioned and NL skin expressed IL-17 and IL-22, even in the absence of additional stimulation (Figure 11B). The expression of IL-17 was significantly increased in the skin lesions of the psoriasis patients both in the absence of additional stimulation and after stimulation compared with the unstimulated CS (Figure 11C). The expression of IL-22 in the psoriasis patients was significantly increased after stimulation compared with the non-stimulated CS (Figure 11D). The percentage of cells that expressed IL-22 and notably IL-17 was increased in the Lin^−^ CD123^low^ population compared with the CD127^+^ population (Figures 11C,D). These findings strongly suggest that SDF-1-dependent migration of Lin^−^ CD123^+^ CD127^low^ cells may precede the activation the downregulation of Lin^−^ CD123 and local production of IL-22 and remarkably IL-17 in psoriasis patients.

## Discussion

In the past years, a high diversity of ILC, including non-classical populations, has been described. Here, we report a Lin^−^ CD123^+^ CD127^low^ population in PB that possesses ILC features. Moreover, IL-3 appears to be crucial for its maintenance and identity. The Lin^−^ CD123^+^ CD127^low^ population highly expresses CLA and has skin-homing potential. Moreover, a similar population CD123^low^ was identified in the skin, which likely participates in the pathogenesis hallmarks of psoriasis.

Recently, different transcription factors have been described as crucial for ILC identity and development. NFIL3, which is regulated by IL-7, is crucial for ILC development (16, 19). Classic ILC express IL-7Rα (CD127), although CD123 (IL-3Rα) is usually used to exclude basophils during ILC identification; this report identified a Lin^−^ CD123^+^ CD127^low^ population that expresses basophil and lymphoid markers but remarkably expresses several ILC features, which appears to be regulated by IL-3.

Classic ILC express CD127 however; recently, it has been reported the presence of non-classical CD127^−^ ILC populations and also an early ILC precursor (EILP) CD127^low^ (8, 9). The Lin^−^ CD123^+^ population we report here shows lymphoid morphology and expresses low levels of CD127. However, to explore the lymphoid origin of such population, we evaluated unproductive DJ rearrangements as a molecular fingerprint of early lymphoid progenitors. There were no apparent rearrangements neither in the CD127^+^ classical ILC nor in the Lin^−^ CD123^+^ CD127^low^ population when compared with B-lymphocytes or in the acute B cell leukemia cell line NAL-M6. Nevertheless, the human ILC progenitor has only been described in tissues (46) and not in bone marrow or PB. Therefore, until now, the molecular features of the human ILC precursor are unknown. As an alternative to determine the lymphoid origin, the expression of CD7 was evaluated. Importantly, the Lin^−^ CD123^+^ CD127^low^ population expressed CD7, which has been reported to be expressed in the CLP and maintained in different lymphoid populations (3). Co-expression of CD127 and CD7 in the Lin^−^ CD123^+^ population suggests lymphoid-related features. However, future studies are necessary to formally prove the lymphoid origin of this population.

CD123 is normally used to exclude pDCs, basophils, and mast cells when identifying ILC. However, the Lin^−^ CD123^+^ CD127^low^ population showed clear differences in the expression of MHC-II, BDCA-4, and BDCA-2 compared to pDCs. With regard to mast cell similarities, the low abundance of mast cells in PB under normal conditions suggest that the CD123^+^ CD127^low^ population are not mast cells or their precursors, as they did not expressed CD34 (35). Nevertheless, in this report, we identified a mixture of lymphocyte-sized cells with no segmented nuclei (no lobes) and cells with classical basophil morphology (lobed nuclei). In addition, two populations of Lin^−^ CD123^+^ cells were observed, the Lin^−^ CD123^+^ CD127^low^ population from the lymphoid region (FSC^low^/SSC^low^) and an FSC^int^ SSC^int^ CD123^+^ population. The Lin^−^ CD123^+^ CD127^low^ CD7^low^ population from the lymphoid region expresses similar levels of the basophil markers FcεR and CCR3, compared to the CD123^+^ FSC^int^ SSC^int^ region. However, it expresses lower levels of CD203c and of the antigen recognized by mAb 2D7, which is expressed in basophil granules, indicating the expression of less granules in this population (43). Our results show then, a distinct Lin^−^ CD123^+^ CD127^low^ population within the lymphoid region with ILC properties that transiently share some features with CD123^+^ basophils from the FSC^int^ SSC^int^ region. Importantly, such population decreases the expression of basophil markers upon activation, whereas most CD123^+^ cells from the FSC^int^ SSC^int^ region maintains the expression of CD123 and show more stable basophil marker display after activation. Of special interest for our future investigations, subfractioning the two subsets described in this study will be highly relevant for further transcriptional analyses at the clonal level. Whether basophils and the Lin^−^ CD123^+^ CD127^low^ population develop from a common progenitor, or their shared phenotypic properties only resemble the phenomenon referred to as lineage priming where “promiscuous expression of several lineage-affiliated genes precedes lineage commitment but does not alter the biological potential” as described for some oligo- or bipotential precursors (47), is still a matter in question.

Remarkably, in this report, several evidences support the finding that the Lin^−^ CD123^+^ CD127^low^ from the lymphoid region population possesses several ILC features. First, the expression of low levels of CD127 by protein and mRNA. Second, the observed expression of CD7, which is related to CLP and maintained in different lymphoid cells, including the classical ILC. Third, the high expression of CD132, which has been reported as crucial for ILC development (48). Fourth, the expression of CD90, a classical ILC marker as well as other ILC markers such as CD161, α4 integrin which has been described in ILC precursors, and the expression of c-Kit, CRTH2, AhR, IL-23R, and CCR6. Fifth, the expression of several transcription factors such as Id2, NFIL3, TOX, PLZF described for ILC identification in humans (16, 17, 34) and TCF-1, recently described for ILC development in mice (15, 49), and sixth, the increase in NFIL3, TOX, and PZLF expression by IL-3 suggesting that NFIL3 might regulate the identity of the ILC-related features in the Lin^−^ CD123^+^ CD127^low^ population. Thus, our data indicate that the use of anti-FcεR and anti-CD123 in the linage cocktail in previous reports may have limited the identification of an alternative population with ILC features in PB.

In human PB, classical ILC express CD161 and exhibit phenotypic diversity (ILC1, ILC2, and ILC3 NKp44^−^). Interestingly, the PB Lin^−^ CD123^+^ CD127^low^ population comprises a majority conspicuous population (CD161^low^, c-Kit^+^, and CRTH2^+^), which, by phenotype, is similar to ILC2 and a minority of ILC1 and ILC3 populations. This finding was inconsistent with the low expression of GATA-3 and the expression of T-bet and RORγt. GATA-3 has been reported as crucial for classical ILC development (50, 51). Moreover, it has recently been reported that a common ILC progenitor RORγt^+^ present in secondary lymphoid tissue has the potential *in vitro* to give rise to all human ILC subpopulations (46). These findings support the idea that the expression of RORγt in the Lin^−^ CD123^+^ CD127^low^ population in PB may be related to a further process of differentiation and diversification of this population in peripheral tissues. In contrast, in the classical CD127^+^ ILC population in PB, similar to other reports (39–41), ILC1, 2, and 3, as well as the expression of T-bet, RORγt, and GATA-3, were identified. These findings indicate that both populations share several ILC features; however, the Lin^−^ CD123^+^ CD127^low^ population in PB may be in a different stage of differentiation and it may require different transcriptional factors and cytokines for development.

It has been reported that ILC express cytokines similar to the Th lymphocytes in peripheral tissues (4, 52). However, there are few reports in PB and most of the studies use cell lines derived from ILC obtained from patients (7, 39, 40). The freshly isolated Lin^−^ CD123^+^ CD127^low^ population expresses only IL-4 after activation and the classical CD127^+^ population expresses IL-2 (data not shown). However, among the total PBMCs, the Lin^−^ CD123^+^ CD127^low^ and the classic CD127^+^ ILC populations expressed IFN-γ in response to PMA/Ionomycin and the ILC1 activation cocktail. However, even in the presence of IL-1β, IL-2, and IL-23, the Lin^−^ CD123^+^ CD127^low^ and CD127^+^ ILC did not express IL-17 or IL-22, which indicates that steady-state PB ILC populations may require further differentiation or activation to express all Th type varieties of cytokines. Importantly, the Lin^−^ CD123^+^ CD127^low^ population highly expresses IL-8 compared with the classical CD127^+^ ILC, notwithstanding that IL-8 expression by ILC has only been assessed in a limited number of reports (6, 53), and the function of this cytokine expression in PB ILC has not been investigated. Interestingly, the population that expresses cytokines after activation shows a downregulation of CD123, therefore further examination of the phenotype and function of the Lin^−^ CD123^+^ CD127^low^ population after activation was evaluated. Interestingly, the population downregulating CD123 expresses IL-8 and decreases the expression of basophil markers, including 2D7. Of note, these effects were not observed in the population that maintains the CD123 expression or after IgE croslinking. However, upon IgE activation, an important decrease in the 2D7 expression was observed in the Lin^−^ CD123^+^ CD127^low^ population, suggesting the activity of this population in response to IgE. These results suggest that activation of the Lin^−^ CD123^+^ CD127^low^ cells may precede their capability of acquiring ILC function and diminishes basophil activity.

In mouse models, it has been proposed that the complete differentiation of ILC occurs in peripheral tissues (15, 49). In human tonsils, the identification of an ILC3 subpopulation that presents a “naïve” phenotype has been recently described; these cells were unresponsive to IL-23 and IL-1β, despite their expression of IL23R and IL1R1 transcripts, and were characterized by the expression of CD62L and CD45RA (23). Our findings demonstrated that the Lin^−^ CD123^+^ CD127^low^ population in PB has limited Th cytokine production and highly expresses homing molecules (CLA, CXCR4, and CD62L) and also a IL23R and AhR, which, in addition to the expression of RORγt, suggests that this population may be in an early stage of differentiation with the potential to migrate into different tissues to be fully differentiated.

Importantly, an equivalent of the CD123^+^ population was present in the CS that expresses several ILC features (CD127^int^, CD132, CD90, c-Kit, CRTH2, AhR, IL-23R, NKp44, and CCR6), where an increased phenotypic diversity (ILC1, ILC2, and ILC3) was also identified. Consistent with this diversity, more Th type cytokines, including IL-22 and remarkably IL-17, were identified in the skin Lin^−^ CD123^+^ population after activation. The expression of IL-22 and IL-17 was consistent with the high expression of IL-23 and AhR (54) in the Lin^−^ CD123^low^ population. This finding suggests that in peripheral tissues, this population may express a wider variety of cytokines as reported for other ILC subsets (24, 28). These findings also support that in the skin, the CD123^+^ population becomes fully differentiated, as proposed in the mouse model for the classical ILC subsets (15, 55). The cytokine expression by the direct isolated skin ILC was not evaluated; therefore, it is possible that other activated cells in the skin cultures may contribute to the cytokine expression by the Lin^−^ CD123^low^ CD127^int^ population, like has been described for ILC2 in skin (56). Remarkably, the Lin^−^ CD123^low^ population in skin expresses IL-8, which may be relevant in the recruitment of other innate cells, such as neutrophils (57). Similar to the PB, the CD123^+^ population in the skin is more frequent than the classical CD127^+^ ILC. Therefore, it is possible that the high expression of CLA may be involved in non-inflammatory skin homing as reported for T cells (58) and ILC, in which CLA expression in ILC2 and ILC3 is sufficient to identify cells with potential to migrate into the skin (39, 40). Our findings suggest that the Lin^−^ CD123^+^ CD127^low^ CLA^+^ population identified in PB may represent a steady-state reservoir with the potential to migrate into the skin, mediated by CLA, and suggest that barrier tissues, such as skin, are normally infiltrated by specialized Lin^−^ CD123^low^ CD127^int^ populations with ILC-related features.

In addition, we demonstrated that the Lin^−^ CD123^+^ CD127^low^ population transmigrates in response to SDF-1 and in the presence of activated endothelial cells mediated by SDF-1. Similar to other reports (59, 60) and other inflammatory diseases (61), an increase in the levels of SDF-1 in the supernatants of psoriasis patients was identified; this finding suggests that the CXCR4/SDF-1-dependent migration is an additional migration mechanism, which may be relevant during inflammatory conditions, such as psoriasis. A CCR10–CCL27 interaction has been proposed as a mechanism for homeostatic skin migration. However, in mouse models of inflammation, a decrease in the number of CCR10^+^ ILC in the skin has been reported (62), in addition to a decreased expression of CCL27 in psoriasis (63). In this report, we propose that CXCR4-SDF-1 is an alternative mechanism in psoriasis (and inflammatory conditions), which may explain the presence of ILC and cytokine production in psoriasis patients even in the absence of CCL27.

Importantly, both the Lin^−^ CD123^low^ and CD127^+^ populations were increased in the skin of psoriasis patients; however, it is possible that the CD127^+^ ILC may migrate by a mechanism independent of CXCR4/SDF-1 but CCR6-dependent because high levels of CCR6 were identified in the CD127^+^ ILC population. Remarkably, both populations express IL-22 and IL-17 in the skin of psoriasis patients and represent two hallmark cytokines in the immunopathology of psoriasis in mouse models and humans (64–66). The expression of these cytokines was increased in the Lin^−^ CD123^low^ population compared with the CD127^+^ ILC. It has been reported that a CD3^−^ population expresses IL-17 in the PB and skin of psoriasis patients (41). However, there is only one report of a slight production of IL-17 by classic NCR^+^ ILC3 from the skin of psoriasis patients (40). Our findings indicate that the Lin^−^ CD123^low^ population may be an important and additional innate source of IL-22 and, importantly, IL-17 in the lesioned and probably in the NL skin of psoriasis patients. In addition, our findings suggest that the SDF-1-dependent migration of Lin^−^ CD123^+^ CD127^low^ cells from the PB to the skin may precede the activation and local production of IL-17 and IL-22 in psoriasis patients.

**Figure 12:**
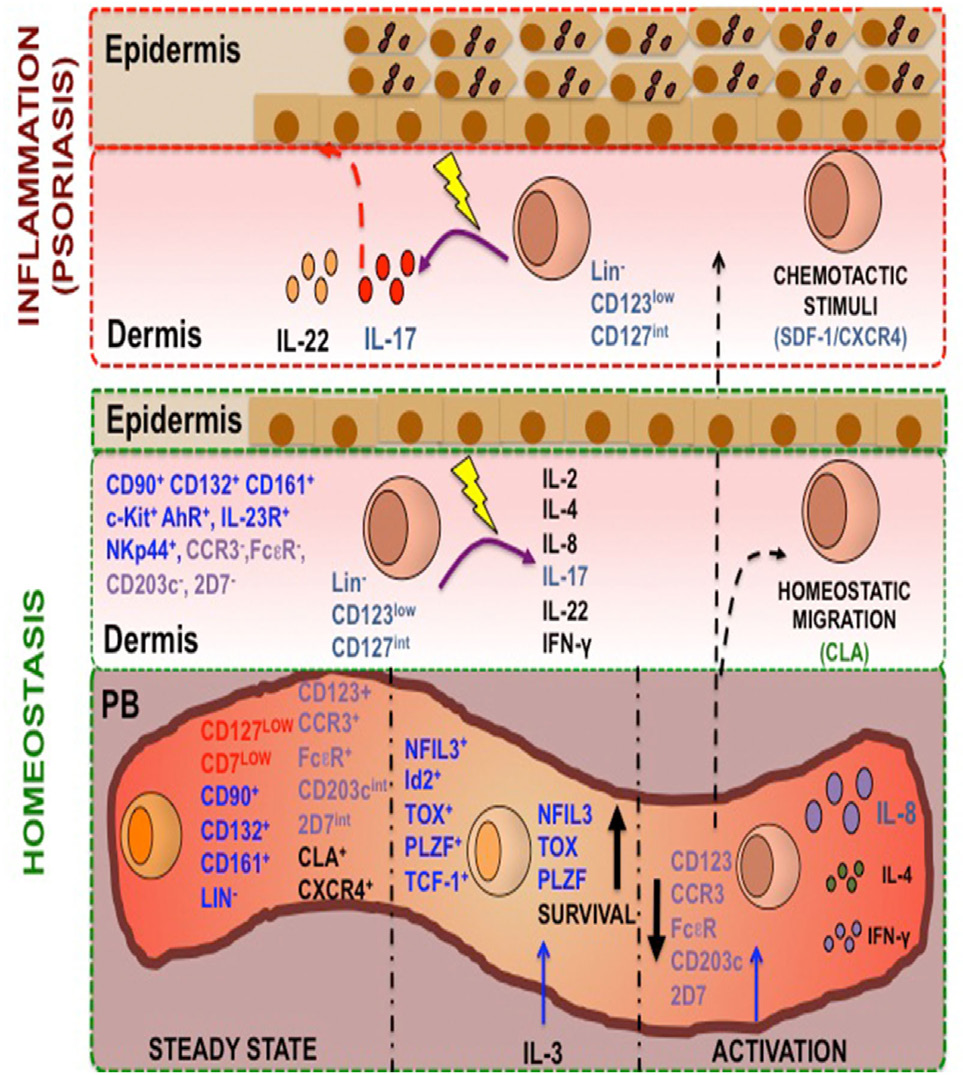
**A human Lin^−^ CD123^+^ CD127^low^ endowed with innate lymphoid cells (ILC) features and migratory capabilities contributes to immunopathological hallmarks of psoriasis**. Peripheral blood (PB) contains a Lin^−^ CD123^+^ population with a mixture lymphoid (in red: CD127^low^ CD7^low^) and basophil markers expression (in purple: FcεR, CCR3, CD203^int^ 2D7^low^) and is endowed with high migratory capabilities [cutaneous lymphocyte antigen (CLA) and CXCR4]. In steady state, this population possesses several ILC features (in blue: Lin^−^, CD132^+^, CD90^+^, CD161^+^, NFIL-3^+^, TCF-1^+^, Id2^+^, TOX^+^, PLZF^+^) and after activation with IL-3 increase this features. This population after PMA/Iono treatment downregulates CD123, is able to produce IL-8, IL-4, and IL-2, and diminish the basophil markers. A similar but specialized CD123^low^ population normally infiltrates barrier tissues, such as skin. We propose that CXCR4-SDF-1 is an important skin-homing mechanism under inflammatory conditions, particularly in psoriasis. The increase of the CD123^low^ population in the non-lesioned and lesioned skin of psoriasis patients supports its high migratory potential. Remarkably, the expression of IL-22 and particularly IL-17 by the CD123^low^ population in the skin of psoriasis patients strongly suggests that this population may contribute to the immunopathological hallmarks of a skin disease such as psoriasis.

In summary, according to the proposed model (Figure 12), we have identified in PB a novel Lin^−^ CD123^+^ CD127^low^ population with a mixture of lymphoid (CD127^low^ CD7^low^) and basophil (FcεR, CCR3, CD203^int^ 2D7^low^) properties that possesses several ILC features, including the phenotype Id2^+^ NFIL3^+^ PLZF^+^ TOX^+^ TCF-1^+^ CD132^+^ CD90^+^ CD161^+^α4 integrin^+^ c-Kit^+^ CRTH2^+^AhR^+^IL-23R^+^ CCR6^+^, and high migratory capabilities. IL-3 appears to comprise a crucial growth factor for survival of the ILC-related features in the Lin^−^ CD123^+^ CD127^low^ population. A similar but specialized Lin^−^ CD123^low^ population normally infiltrates barrier tissues, such as skin. We propose that CXCR4/SDF-1 is an important skin-homing mechanism under inflammatory conditions in psoriasis. The increase of the Lin^−^ CD123^low^ population in the NL and lesioned skin of psoriasis patients supports its high migratory potential. Remarkably, expression of IL-22 and particularly IL-17 by the Lin^−^ CD123^low^ population in the skin of psoriasis patients strongly suggests that this population may contribute to the immunopathological hallmarks of a skin disease such as psoriasis.

## Ethics Statement

The study was approved by the local ethic committee from the Centro Dermatologico Ladislao de la Pascua Registry number: 112/2016 and by the ethic commitee for Health Research from Instituto Mexicano del Seguro Social (IMSS) (commitee number:3601). The study was conducted according to the principles detailed in the Declaration of Helsinki. All participants signed an informed consent form.

## Author Contributions

LB conceived and directed the project. LB, LM-V, and OC-E designed the experiments. LM-V, OC-E, AM, CA-F, and MV-A performed the experiments. LM-V and OC-E acquired and analyzed the data. LB and RP contributed reagents/materials/analysis tools. LB, LM-V, and RP wrote the manuscript. CM-G and FJ-S were involved in the recruitment and diagnosis of psoriasis patients. EF-O provided the control skin biopsies. M-TL realized the Quantitative RT-PCR and JM-B and JT-S realized the incomplete DH-JH rearrangements analysis. All the authors reviewed critically the manuscript.

## Conflict of Interest Statement

The authors declare that the research was conducted in the absence of any commercial or financial relationships that could be construed as a potential conflict of interest.

## Abbreviations

AhR, aryl hydrocarbon receptor; BCR, B cell receptor; BDCA, blood dendritic cell antigen; cDC, conventional dendritic cells; CLA, cutaneous lymphocyte antigen; CLP, common lymphoid precursor; CRTH2, chemoattractant receptor-homologous molecule expressed on Th2 cells or prostaglandin D_2_ receptor 2; CS, control skin; GATA-3, trans-acting T-cell-specific transcription factor; HD, healthy donors; HLA-DR, human leukocyte antigen-antigen D related; HUVEC, human umbilical vein endothelial cells; ILC, innate lymphoid cells; NCR, natural cytotoxicity receptors; NFIL3, nuclear factor, IL-3 regulated; NK, natural killer; PB, peripheral blood; PBMCs, peripheral blood mononuclear cells; pDCs, plasmacytoid dendritic cells; PLZF, promyelocytic leukemia zinc finger; PMA, phorbol 12-myristate 13-acetate; ROR-γt, RAR-related orphan receptor gamma; SDF-1, stromal cell-derived factor-1; T-bet, T-box transcription factor; TCR, T cell receptor; TCF-1, T cell factor-1; Th, T helper; TOX, thymocyte selection-associated high-mobility group box protein.
