# Supplementary material for: A Human Lin− CD123+ CD127low Population Endowed with ILC Features and Migratory Capabilities Contributes to Immunopathological Hallmarks of Psoriasis
Source: Front Immunol. 2017 Mar 2;8:176. doi: 10.3389/fimmu.2017.00176 (PMC5332395; doi:10.3389/fimmu.2017.00176)
Supplement: Supplementary file 1 [file Data_Sheet_1.pdf]

Supplementary Material

A HUMAN LIN- CD123+ CD127low POPULATION ENDOWED WITH ILC FEATURES AND MIGRATORY CAPABILITIES CONTRIBUTES TO IMMUNOPATHOLOGICAL HALLMARKS OF PSORIASIS

Mora-Velandia Luz1,2, Castro-Escamilla Octavio1,2, González-Méndez Andrés1, Aguilar-Flores Cristina1, Velázquez-Avila Martha4, Tussié-Luna María Isabel5,6, Jesus Martínez-Barnetche7, Juan Téllez-Sosa7, Maldonado César3, Jurado-Santacruz Fermín3, Ferat-Osorio Eduardo1, Pelayo Rosana4 and Bonifaz Laura1*.

1.Unidad de Investigación Médica en Inmunoquímica Hospital de Especialidades Centro Médico Nacional Siglo XXI. Instituto Mexicano del Seguro Social. Mexico city, Mexico.
2. Universidad Nacional Autónoma de México (UNAM). Mexico city, Mexico.
3. Centro Dermatológico "Dr. Ladislao de la Pascua", Secretaria de Salud de la Ciudad de México. Mexico city, Mexico.
4. Unidad de Investigación en Enfermedades Oncológicas, Hospital de Oncología, Centro Médico Nacional 'Siglo XXI'. Mexico city, Mexico.
5. División de Investigación; Facultad de Medicina; Universidad Nacional Autónoma de México (UNAM). Mexico city, Mexico.
6. Unidad de Investigación en Virología y Cáncer; Hospital Infantil de México "Federico Gómez". Mexico city, Mexico.
7. Centro de Investigación Sobre Enfermedades Infecciosas. Instituto Nacional de Salud Pública. Cuernvaca, Morelos México

Correspondence:
Laura Bonifaz
e-mail: labonifaz@yahoo.com

Suplementary Figure 1

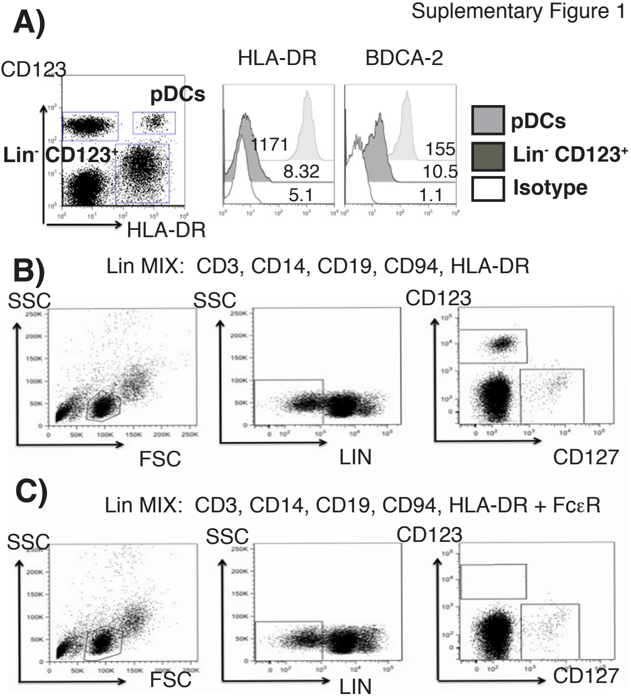

Supplementary Figure 1: In A) Gate analysis on the Lin- CD123+ and pDCs, and compared expression of HLA-DR and BDCA-2 in both populations B) and C): Identification of Lin- CD123+ and Lin- CD127+ cells using -/+ anti-FceR in the linage cocktail respectively.

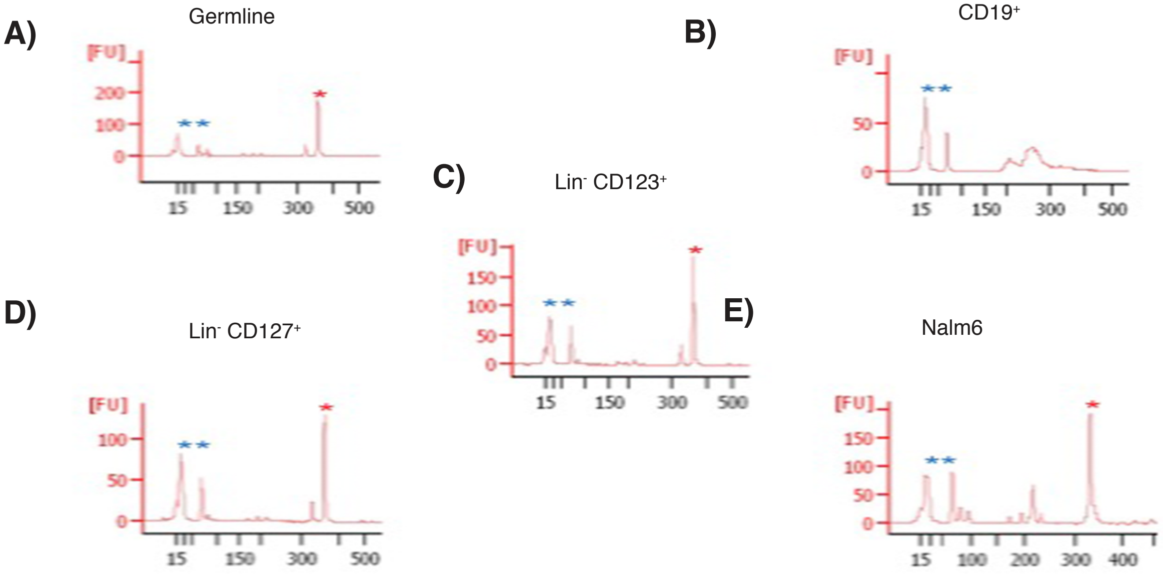

Supplementary Figure 2: Supplementary figure X: DH1-6-JH incomplete rearrangement analysis. Genomic DNA from each subpopulation was subjected to multiplexed PCR amplification using the BIOMED-2 primers set for DH1-6-JH incomplete rearrangements (Tube D) according to (Ref 40). PCR products were resolved in a Agilent 2100 Bioanalyzer capillary electrophoresis system with an 1000 DNA chip. Product size in base pairs in the x-axis (cropped for clarity) and relative amount (fluorescence units, y-axis). A) Oral swab from a healthy donor as a negative control; B) Sorted CD19+ B cells from a healthy donor; C) Sorted CD123+ CD127low from a healthy donor; D) CD127+ classical ILC from a healthy donor; and E) Acute B- cell leukemia cell line NAL-M6. The expected range of DH1-6-JH rearrangements is 110-420 bp (red bar). A germline ~340 bp product (*) is consistently amplified and serves as an internal positive control. Low weight electrophoretic front (**).

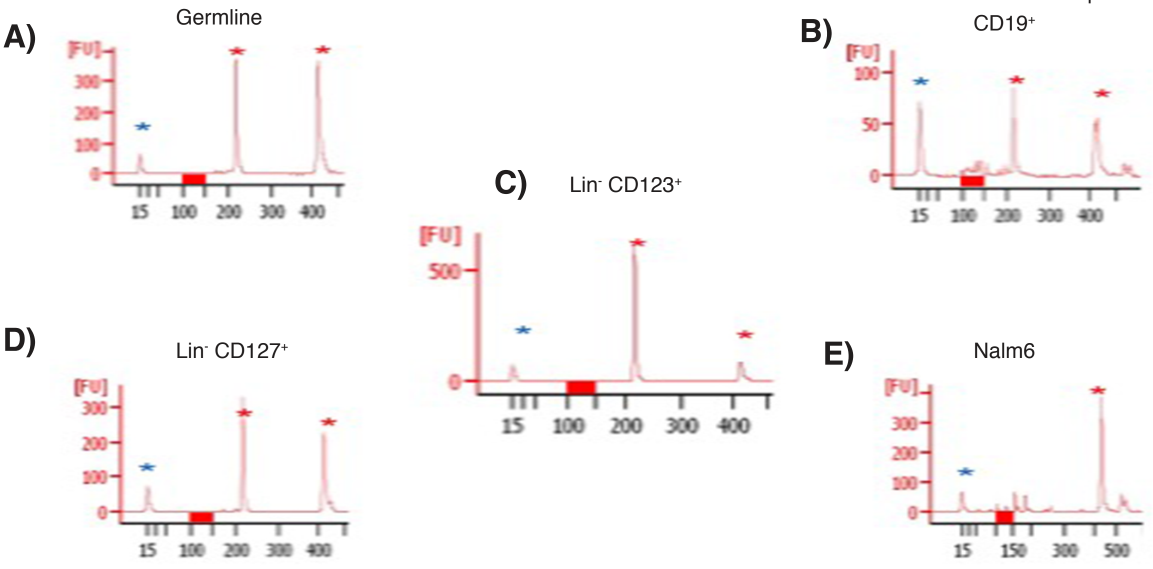

Supplementary Figure 3:DH7-JH incomplete rearrangement analysis. Genomic DNA from each subpopulation was subjected to PCR amplification using the BIOMED-2 DH7-JH primer pair for incomplete rearrangements (Tube E) according to (Ref1). PCR products were resolved in a Agilent Bioanalyzer capillary electrophoresis system with an 1000 DNA chip. Product size in base pairs in the x-axis (cropped for clarity) and relative amount (fluorescence units, y-axis). A) Oral swab from a healthy donor as a negative control; B) Sorted CD19+ B cells from a healthy donor; C) Sorted CD123+ CD127low from a healthy donor; D) CD127+ classical ILC from a healthy donor; and E) Acute B- cell leukemia cell line NAL-M6. The expected range of DH7-JH rearrangements is 100-130 bp (red bar). Two germline ~211 and 419 bp products (*) are consistently amplified and serve as internal positive controls. Low weight electrophoretic front (*).

Suplementary Figure 4

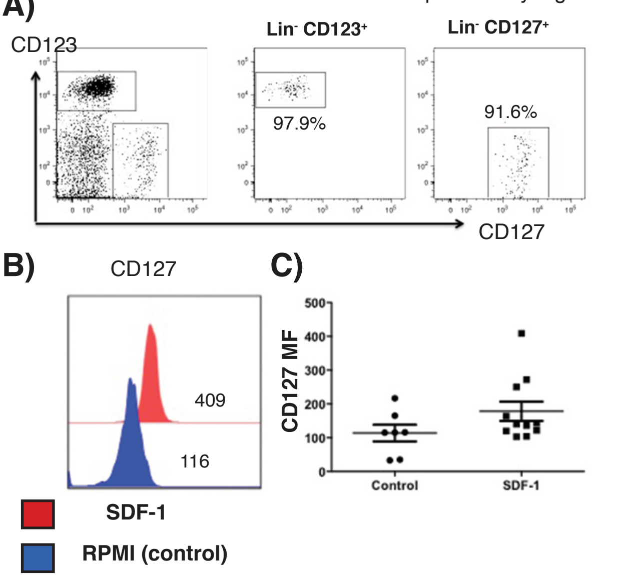

Supplementary Figure 4: In **A)** Percentages of Lin$^-$ CD123$^+$ and Lin$^-$ CD127$^+$ sorted cells. Gated on Lin$^-$ cells. **B)** CD127 expression in Lin$^-$ CD123$^+$ after 3.5 hrs of transmigration assay in response SDF-1(red histogram) or RPMI (blue hisrogram). MF values representative of three experiments are shown. C) Graph of CD127 MF from three independent experiments. Control: n = 6 and SDF-1: n= 11

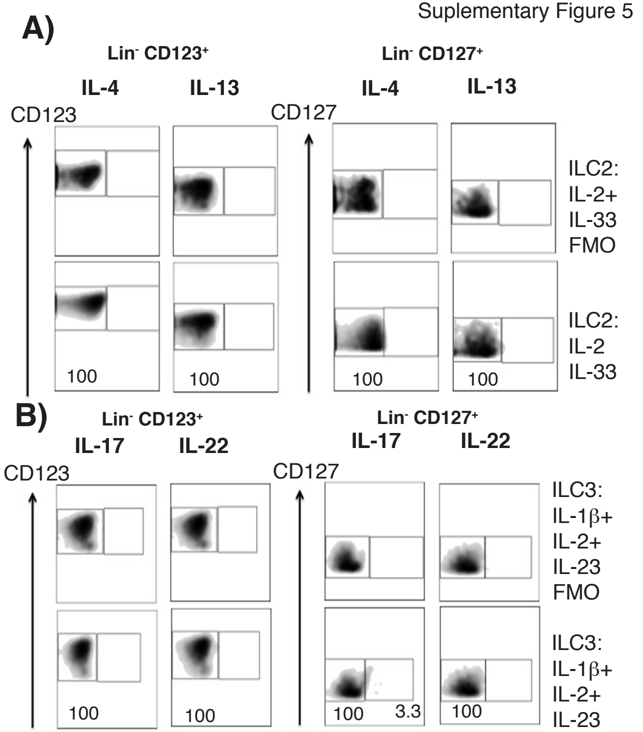

Supplementary Figure 5: A) IL-4 and IL-13 expression in Lin- CD123+ and Lin- CD127+ within PBMCs cultured +/- ILC2 activation cocktail for 18 hrs. and B): IL-17 and IL-22 expression in Lin- CD123+ and Lin- CD127+ within PBMCs cultured +/- ILC3 activation cocktail for 18 hrs. Density plots are representative of at least three independent experiments

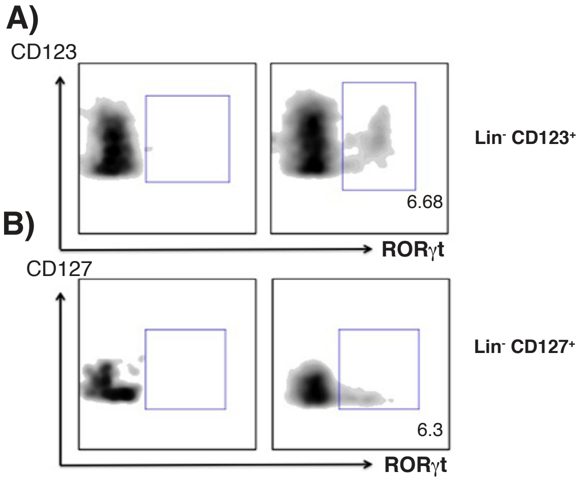

Supplementary Figure 6:
RORγt expression in total skin cells in A) Lin⁻ CD123⁺ and B) Lin⁻ CD127⁺ cell populations . Left: FMO controls, Right: RORγt staining
